# Supplementary material for: Assessing quality of life in psychosocial and mental health disorders in children: a comprehensive overview and appraisal of generic health related quality of life measures
Source: BMC Pediatr. 2020 Jul 3;20:329. doi: 10.1186/s12887-020-02220-8 (PMC7333319; doi:10.1186/s12887-020-02220-8)
Supplement: Supplementary file 1 — Additional file 1: Appendix 1. Search terms instruments. Appendix 2. Search terms psychometric quality. Appendix 3. Cosmin Definitions. Appendix 4. Quality scores Questionnaires. Appendix 5. PRISMA flow charts Review of reviews. Appendix 6. Prisma Flow chart Psychometric characteristics. Appendix 7. Summary Tables of psychometric research. Appendix 8. Domains of QoL per age group. [file 12887_2020_2220_MOESM1_ESM.docx]

# Supplements

Index

[Supplements 1](#_Toc37860025)

[Appendix 1 Search terms instruments 2](#_Toc37860026)

[Appendix 2. Search terms psychometric quality 6](#_Toc37860027)

[Appendix 3. Cosmin Definitions 15](#_Toc37860028)

[Appendix 4. Quality scores Questionnaires. 19](#_Toc37860029)

[Appendix 5. PRISMA flow charts Review of reviews 24](#_Toc37860030)

[Appendix 6. Prisma Flow chart Psychometric characteristics 26](#_Toc37860031)

[Appendix 7 Summary Tables of psychometric research 49](#_Toc37860032)

[Table 1. 16D 49](#_Toc37860033)

[Table 2. 17d 50](#_Toc37860034)

[Table 3. AQOL 51](#_Toc37860035)

[Table 4. Chip 54](#_Toc37860036)

[Table 5. CHQ 60](#_Toc37860037)

[Tabel 6. Comprehensive health status classification system -preschool 71](#_Toc37860038)

[Tabel 7. CHUd9 72](#_Toc37860039)

[Tabel 8. Child quality of life questionnaire (CQOL) 76](#_Toc37860040)

[Table 9. EQ-5D 77](#_Toc37860041)

[Tabel 10. Generic children’s quality of life questionnaire (GCQ) 82](#_Toc37860042)

[Table 11. HUI 83](#_Toc37860043)

[Tabel 12. ITQOL 84](#_Toc37860044)

[Table13. Kidscreen 85](#_Toc37860045)

[Table 14. KINDL-R 98](#_Toc37860046)

[Tabel 15. Multidimensional students life satisfaction scale 104](#_Toc37860047)

[Tablel16. PEDSQL 106](#_Toc37860048)

[Tabel 17. QOLPAV 124](#_Toc37860049)

[Tabel 18. Quality of well being scale – mental health subscale 125](#_Toc37860050)

[Tabel 19. TNO-AZL child quality of life (TACQOL) 126](#_Toc37860051)

[Table 20. TAPQOL 127](#_Toc37860052)

[Table 21. Youth quality of life instrument (YQOL) 130](#_Toc37860053)

[Appendix 8. Domains of QoL per age group 131](#_Toc37860054)

[Table Domains of QoL 0-8 years 131](#_Toc37860055)

[Table Domains of QoL 9-12 years 132](#_Toc37860056)

[Table Domains of QoL 13-18 years 133](#_Toc37860057)

# Appendix 1 Search terms instruments

Pubmed (Medline)

(("Neurodevelopmental Disorders"[Mesh] OR "Anxiety Disorders"[Mesh] OR "Disruptive, Impulse Control, and Conduct Disorders"[Mesh] OR neurodevelopmental[tiab] OR ADHD[tiab] OR ADD[tiab] OR attention deficit*[tiab] OR hyperactiv*[tiab] OR hyperkinetic*[tiab] OR minimal brain deficit*[tiab] OR minimal brain dysfunction*[tiab] OR anxiet*[tiab] OR affective disorder*[tiab] OR affective psychos*[tiab] OR mood disorder*[tiab] OR psychotic depressi*[tiab] OR bipolar disorder*[tiab] OR depressive disorder*[tiab] OR depression*[tiab] OR fobia*[tiab] OR phobia*[tiab] OR phobic*[tiab] OR obsessive compulsive[tiab] OR Pervasive developmental disorder*[tiab] OR Asperger*[tiab] OR PDD NOS[tiab] OR autism*[tiab] OR autist*[tiab] OR Conduct disorder*[tiab] OR Oppositional defiant disorder*[tiab] OR CD[tiab] OR ODD[tiab] OR Disruptive Behavio*[tiab] OR oppositional behavio*[tiab] OR conduct problems[tiab] OR Impulse Control Disorder*[tiab] OR Reading Disorder*[tiab] OR Language disorder*[tiab] OR learning disabilit*[tiab] OR learning disorder*[tiab] OR Dyslexia[tiab] OR Word Blindness*[tiab] OR dyscalculia[tiab] OR childhood-onset-fluency disorder*[tiab] OR stuttering[tiab] OR speech sound disorder*[tiab] OR communication disorder*[tiab] OR Developmental Coordination Disorder*[tiab] OR Stereotypic Movement Disorder*[tiab] OR Body Rocking[tiab] OR Body-Focused Repetitive Behavio*[tiab] OR trichotillomania[tiab] OR Tic Disorder*[tiab] OR Tourette syndrome*[tiab] OR Gilles de la Tourette*[tiab])

AND

(child*[tw] OR schoolchild*[tw] OR infan*[tw] OR adolescen*[tw] OR pediatri*[tw] OR paediatr*[tw] OR boy[tw] OR boys[tw] OR boyhood[tw] OR girl[tw] OR girls[tw] OR girlhood[tw] OR youth[tw] OR youths[tw] OR teen[tw] OR teens[tw] OR teenager*[tw] OR puberty[tw] OR preschool*[tw] OR toddler*[tw] OR juvenile*[tw] OR ‘high school’[tw] or kindergarden[tw])

AND

(review*[tiab] OR meta-analys*[tiab] or systematic review*[tiab] OR sysrev_methods [sb])

AND

(((((Quality of life[tiab] OR well-being[tiab] OR wellbeing[tiab] OR QoL[tiab]) AND (measurement*[tiab] OR assessment*[tiab] OR instrument*[tiab] OR questionnaire*[tiab])) or (QALY[tiab] or quality adjusted life year*[tiab] or 'health related quality of life'[tiab]))))

Results 8/9/2017: 403

Psycinfo

DE "Oppositional Defiant Disorder" OR DE "Attention Deficit Disorder" OR DE "Attention Deficit Disorder with Hyperactivity" OR DE "Attention Deficit Disorder with Hyperactivity" OR DE "Conduct Disorder" OR DE "Anxiety Disorders" OR DE "Generalized Anxiety Disorder" OR DE "Obsessive Compulsive Disorder" OR DE "Panic Disorder" OR DE "Phobias" OR DE "Acrophobia" OR DE "Agoraphobia" OR DE "Claustrophobia" OR DE "Ophidiophobia" OR DE "School Phobia" OR DE "Social Phobia" OR DE "Posttraumatic Stress Disorder" OR DE "Separation Anxiety" OR DE "Impulse Control Disorders" OR DE "Explosive Disorder" OR DE "Neurodevelopmental Disorders" OR DE "Pervasive Developmental Disorders" OR DE "Aspergers Syndrome" OR DE "Autism" OR DE "Rett Syndrome" OR DE "Developmental Disabilities" OR DE "Specific Language Impairment" OR DE "Communication Disorders" OR DE "Language Disorders" OR DE "Aphasia" OR DE "Echolalia" OR DE "Mutism" OR DE "Specific Language Impairment" OR DE "Speech Disorders" OR DE "Articulation Disorders" OR DE "Dysphonia" OR DE "Stuttering" OR DE "Tics" OR DE "Tourette Syndrome" OR DE "Dyspraxia" OR TI (neurodevelopmental OR ADHD OR ADD OR "attention deficit*" OR hyperactiv* OR hyperkinetic* OR "minimal brain deficit*" OR "minimal brain dysfunction*" OR anxiet* OR "affective disorder*" OR "affective psychos*" OR "mood disorder*" OR "psychotic depressi*" OR "bipolar disorder*" OR "depressive disorder*" OR depression* OR fobia* OR phobia* OR phobic* OR "obsessive compulsive" OR "Anankastic Personalit*" OR "Pervasive developmental disorder*" OR Asperger* OR (PDD AND NOS) OR autism* OR autist* OR "Conduct disorder*" OR "Oppositional defiant disorder*" OR "CD" OR ODD OR "Disruptive Behavio*" OR "oppositional behavio*" OR "conduct problems" OR "Impulse Control Disorder*" OR "Reading Disorder*" OR "Language disorder*" OR "learning disabilit*" OR "learning disorder*" OR Dyslexia OR "Word Blindness*" OR dyscalculia OR "childhood-onset-fluency disorder*" OR stuttering OR "speech sound disorder*" OR "communication disorder*" OR "Developmental Coordination Disorder*" OR "Motor Skills Disorder*" OR "childhood dyspraxia" OR "developmental disorder of motor function*" OR "clumsy child syndrome*" OR "motor development disorder*" OR "Stereotypic Movement Disorder*" OR "Body Rocking" OR "Body-Focused Repetitive Behavio*" OR "Head Banging" OR trichotillomania OR "Tic Disorder*" OR "Tourette syndrome*" OR "Gilles de la Tourette*" OR)) OR AB (neurodevelopmental OR ADHD OR ADD OR "attention deficit*" OR hyperactiv* OR hyperkinetic* OR "minimal brain deficit*" OR "minimal brain dysfunction*" OR anxiet* OR "affective disorder*" OR "affective psychos*" OR "mood disorder*" OR "psychotic depressi*" OR "bipolar disorder*" OR "depressive disorder*" OR depression* OR fobia* OR phobia* OR phobic* OR "obsessive compulsive" OR "Anankastic Personalit*" OR "Pervasive developmental disorder*" OR Asperger* OR (PDD AND NOS) OR autism* OR autist* OR "Conduct disorder*" OR "Oppositional defiant disorder*" OR "CD" OR ODD OR "Disruptive Behavio*" OR "oppositional behavio*" OR "conduct problems" OR "Impulse Control Disorder*" OR "Reading Disorder*" OR "Language disorder*" OR "learning disabilit*" OR "learning disorder*" OR Dyslexia OR "Word Blindness*" OR dyscalculia OR "childhood-onset-fluency disorder*" OR stuttering OR "speech sound disorder*" OR "communication disorder*" OR "Developmental Coordination Disorder*" OR "Motor Skills Disorder*" OR "Stereotypic Movement Disorder*"))

AND

TI (child* OR schoolchild* OR infan* OR adolescen* OR pediatri* OR paediatr* OR boy OR boys OR boyhood OR girl OR girls OR girlhood OR youth OR youths OR teen OR teens OR teenager* OR puberty OR preschool* OR toddler* OR juvenile* OR “high school” or kindergarden) OR AB (child* OR schoolchild* OR infan* OR adolescen* OR pediatri* OR paediatr* OR boy OR boys OR boyhood OR girl OR girls OR girlhood OR youth OR youths OR teen OR teens OR teenager* OR puberty OR preschool* OR toddler* OR juvenile* OR “high school” or kindergarden) OR AB (child* OR schoolchild* OR infan* OR adolescen* OR pediatri* OR paediatr* OR boy OR boys OR boyhood OR girl OR girls OR girlhood OR youth OR youths OR teen OR teens OR teenager* OR puberty OR preschool* OR toddler* OR juvenile* OR “high school” or kindergarden) OR AB (child* OR schoolchild* OR infan* OR adolescen* OR pediatri* OR paediatr* OR boy OR boys OR boyhood OR girl OR girls OR girlhood OR youth OR youths OR teen OR teens OR teenager* OR puberty OR preschool* OR toddler* OR juvenile* OR “high school” or kindergarden)

(TI (review* OR “meta-analys*” or systematic review*) OR AB (review* OR “meta-analys*” or systematic review*)

AND

AB (((Quality of life OR well-being OR wellbeing OR QoL) AND (measurement* OR assessment* OR instrument* OR questionnaire*)) or (QALY or quality adjusted life year* or 'health related quality of life')) OR TI (((Quality of life OR well-being OR wellbeing OR QoL) AND (measurement* OR assessment* OR instrument* OR questionnaire*)) or (QALY or quality adjusted life year* or 'health related quality of life'))

Results 8/9/2017: 260

EMBASE

('behavior disorder'/exp OR 'anxiety disorder'/exp OR 'impulse control disorder'/exp OR 'tic'/exp OR 'developmental coordination disorder'/exp OR neurodevelopmental:ab,ti OR adhd:ab,ti OR add:ab,ti OR (attention NEXT/1 deficit*):ab,ti OR hyperactiv*:ab,ti OR hyperkinetic*:ab,ti OR ('minimal brain' NEXT/1 deficit*):ab,ti OR ('minimal brain' NEXT/1 dysfunction*):ab,ti OR anxiet*:ab,ti OR (affective NEXT/1 disorder*):ab,ti OR (affective NEXT/1 psychos*):ab,ti OR (mood NEXT/1 disorder*):ab,ti OR (psychotic NEXT/1 depressi*):ab,ti OR (bipolar NEXT/1 disorder*):ab,ti OR (depressive NEXT/1 disorder*):ab,ti OR depression*:ab,ti OR fobia*:ab,ti OR phobia*:ab,ti OR phobic*:ab,ti OR 'obsessive compulsive':ab,ti OR (anankastic NEXT/1 personalit*):ab,ti OR ('pervasive developmental' NEXT/1 disorder*):ab,ti OR asperger*:ab,ti OR 'pdd nos':ab,ti OR autism*:ab,ti OR autist*:ab,ti OR (conduct NEXT/1 disorder*):ab,ti OR ('oppositional defiant' NEXT/1 disorder*):ab,ti OR cd:ab,ti OR odd:ab,ti OR (disruptive NEXT/1 behavio*):ab,ti OR (oppositional NEXT/1 behavio*):ab,ti OR 'conduct problems':ab,ti OR ('impulse control' NEXT/1 disorder*):ab,ti OR (reading NEXT/1 disorder*):ab,ti OR (language NEXT/1 disorder*):ab,ti OR (learning NEXT/1 disabilit*):ab,ti OR (learning NEXT/1 disorder*):ab,ti OR dyslexia:ab,ti OR (word NEXT/1 blindness*):ab,ti OR dyscalculia:ab,ti OR ('childhood-onset-fluency' NEXT/1 disorder*):ab,ti OR stuttering:ab,ti OR ('speech sound' NEXT/1 disorder*):ab,ti OR (communication NEXT/1 disorder*):ab,ti OR ('stereotypic movement' NEXT/1 disorder*):ab,ti OR 'body rocking':ab,ti OR ('body-focused repetitive' NEXT/1 behavio*):ab,ti OR 'head banging':ab,ti OR trichotillomania:ab,ti OR (tic NEXT/1 disorder*):ab,ti OR (tourette NEXT/1 syndrome*):ab,ti OR ('gilles de la' NEXT/1 tourette*):ab,ti)

AND

(child*:ab,ti OR schoolchild*:ab,ti OR infan*:ab,ti OR adolescen*:ab,ti OR pediatri*:ab,ti OR paediatr*:ab,ti OR boy:ab,ti OR boys:ab,ti OR boyhood:ab,ti OR girl:ab,ti OR girls:ab,ti OR girlhood:ab,ti OR youth:ab,ti OR youths:ab,ti OR teen:ab,ti OR teens:ab,ti OR teenager*:ab,ti OR puberty:ab,ti OR preschool*:ab,ti OR toddler*:ab,ti OR juvenile*:ab,ti)

AND

(review*:ab,ti OR meta-analys*:ab,ti or ‘systematic review*’:ab,ti OR 'systematic review'/exp)

AND

Quality of life instrument:ab,ti OR well-being:ab,ti OR wellbeing:ab,ti OR QoL:ab,ti OR Quality adjusted life year:ab,ti OR health related quality of life:ab,ti OR QALY:ab,ti

Results 8/9/2017: 423

2.1.5 Econlit

TI (neurodevelopmental OR ADHD OR ADD OR "attention deficit*" OR hyperactiv* OR hyperkinetic* OR "minimal brain deficit*" OR "minimal brain dysfunction*" OR anxiet* OR "affective disorder*" OR "affective psychos*" OR "mood disorder*" OR "psychotic depressi*" OR "bipolar disorder*" OR "depressive disorder*" OR depression* OR fobia* OR phobia* OR phobic* OR "obsessive compulsive" OR "Anankastic Personalit*" OR "Pervasive developmental disorder*" OR Asperger* OR (PDD AND NOS) OR autism* OR autist* OR "Conduct disorder*" OR "Oppositional defiant disorder*" OR "CD" OR ODD OR "Disruptive Behavio*" OR "oppositional behavio*" OR "conduct problems" OR "Impulse Control Disorder*" OR "Reading Disorder*" OR "Language disorder*" OR "learning disabilit*" OR "learning disorder*" OR Dyslexia OR "Word Blindness*" OR dyscalculia OR "childhood-onset-fluency disorder*" OR stuttering OR "speech sound disorder*" OR "communication disorder*" OR "Developmental Coordination Disorder*" OR "Motor Skills Disorder*" OR "childhood dyspraxia" OR "developmental disorder of motor function*" OR "clumsy child syndrome*" OR "motor development disorder*" OR "Stereotypic Movement Disorder*" OR "Body Rocking" OR "Body-Focused Repetitive Behavio*" OR "Head Banging" OR trichotillomania OR "Tic Disorder*" OR "Tourette syndrome*" OR "Gilles de la Tourette*" OR)) OR AB (neurodevelopmental OR ADHD OR ADD OR "attention deficit*" OR hyperactiv* OR hyperkinetic* OR "minimal brain deficit*" OR "minimal brain dysfunction*" OR anxiet* OR "affective disorder*" OR "affective psychos*" OR "mood disorder*" OR "psychotic depressi*" OR "bipolar disorder*" OR "depressive disorder*" OR depression* OR fobia* OR phobia* OR phobic* OR "obsessive compulsive" OR "Anankastic Personalit*" OR "Pervasive developmental disorder*" OR Asperger* OR (PDD AND NOS) OR autism* OR autist* OR "Conduct disorder*" OR "Oppositional defiant disorder*" OR "CD" OR ODD OR "Disruptive Behavio*" OR "oppositional behavio*" OR "conduct problems" OR "Impulse Control Disorder*" OR "Reading Disorder*" OR "Language disorder*" OR "learning disabilit*" OR "learning disorder*" OR Dyslexia OR "Word Blindness*" OR dyscalculia OR "childhood-onset-fluency disorder*" OR stuttering OR "speech sound disorder*" OR "communication disorder*" OR "Developmental Coordination Disorder*" OR "Motor Skills Disorder*" OR "Stereotypic Movement Disorder*"))

AND

TI (child* OR schoolchild* OR infan* OR adolescen* OR pediatri* OR paediatr* OR boy OR boys OR boyhood OR girl OR girls OR girlhood OR youth OR youths OR teen OR teens OR teenager* OR puberty OR preschool* OR toddler* OR juvenile* OR “high school” or kindergarden) OR AB (child* OR schoolchild* OR infan* OR adolescen* OR pediatri* OR paediatr* OR boy OR boys OR boyhood OR girl OR girls OR girlhood OR youth OR youths OR teen OR teens OR teenager* OR puberty OR preschool* OR toddler* OR juvenile* OR “high school” or kindergarden) OR AB (child* OR schoolchild* OR infan* OR adolescen* OR pediatri* OR paediatr* OR boy OR boys OR boyhood OR girl OR girls OR girlhood OR youth OR youths OR teen OR teens OR teenager* OR puberty OR preschool* OR toddler* OR juvenile* OR “high school” or kindergarden) OR AB (child* OR schoolchild* OR infan* OR adolescen* OR pediatri* OR paediatr* OR boy OR boys OR boyhood OR girl OR girls OR girlhood OR youth OR youths OR teen OR teens OR teenager* OR puberty OR preschool* OR toddler* OR juvenile* OR “high school” or kindergarden)

AND

(TI (review* OR “meta-analys*” or systematic review*) OR AB (review* OR “meta-analys*” or systematic review*)

AND

AB (((Quality of life OR well-being OR wellbeing OR QoL) AND (measurement* OR assessment* OR instrument* OR questionnaire*)) or (QALY or quality adjusted life year* or 'health related quality of life')) OR TI (((Quality of life OR well-being OR wellbeing OR QoL) AND (measurement* OR assessment* OR instrument* OR questionnaire*)) or (QALY or quality adjusted life year* or 'health related quality of life'))

Results 8/9/2017: 2

Web of Science

(TS=(neurodevelopmental OR ADHD OR ADD OR "attention deficit*" OR hyperactiv* OR hyperkinetic* OR "minimal brain deficit*" OR "minimal brain dysfunction*" OR anxiet* OR "affective disorder*" OR "affective psychos*" OR "mood disorder*" OR "psychotic depressi*" OR "bipolar disorder*" OR "depressive disorder*" OR depression* OR fobia* OR phobia* OR phobic* OR "obsessive compulsive" OR "Anankastic Personalit*" OR "Pervasive developmental disorder*" OR Asperger* OR (PDD AND NOS) OR autism* OR autist* OR "Conduct disorder*" OR "Oppositional defiant disorder*" OR "CD" OR ODD OR "Disruptive Behavio*" OR "oppositional behavio*" OR "conduct problems" OR "Impulse Control Disorder*" OR "Reading Disorder*" OR "Language disorder*" OR "learning disabilit*" OR "learning disorder*" OR Dyslexia OR "Word Blindness*" OR dyscalculia OR "childhood-onset-fluency disorder*" OR stuttering OR "speech sound disorder*" OR "communication disorder*" OR "Developmental Coordination Disorder*" OR "Motor Skills Disorder*" OR "childhood dyspraxia" OR "developmental disorder of motor function*" OR "clumsy child syndrome*" OR "motor development disorder*" OR "Stereotypic Movement Disorder*" OR "Body Rocking" OR "Body-Focused Repetitive Behavio*" OR "Head Banging" OR trichotillomania OR "Tic Disorder*" OR "Tourette syndrome*" OR "Gilles de la Tourette*"))

AND

(TS=(child* OR school* OR infan* OR adolescen* OR pediatri* OR paediatr* OR boy OR boys OR boyhood OR girl OR girls OR girlhood OR youth OR youths OR teen OR teens OR teenager* OR puberty OR preschool* OR toddler* OR juvenile* OR kids))

AND

(TS=( review* OR “meta-analys*” or “systematic review*”))

AND

(TS=( “Quality of life instrument” OR well-being OR wellbeing OR QoL OR “Quality adjusted life year” OR “health related quality of life” OR QALY))

Results 8/9/2017: 408

Cochrane

(neurodevelopmental OR ADHD OR ADD OR "attention deficit*" OR hyperactiv* OR hyperkinetic* OR "minimal brain deficit*" OR "minimal brain dysfunction*" OR anxiet* OR "affective disorder*" OR "affective psychos*" OR "mood disorder*" OR "psychotic depressi*" OR "bipolar disorder*" OR "depressive disorder*" OR depression* OR fobia* OR phobia* OR phobic* OR "obsessive compulsive" OR "Anankastic Personalit*" OR "Pervasive developmental disorder*" OR Asperger* OR (PDD AND NOS) OR autism* OR autist* OR "Conduct disorder*" OR "Oppositional defiant disorder*" OR "CD" OR ODD OR "Disruptive Behavio*" OR "oppositional behavio*" OR "conduct problems" OR "Impulse Control Disorder*" OR "Reading Disorder*" OR "Language disorder*" OR "learning disabilit*" OR "learning disorder*" OR Dyslexia OR "Word Blindness*" OR dyscalculia OR "childhood-onset-fluency disorder*" OR stuttering OR "speech sound disorder*" OR "communication disorder*" OR "Developmental Coordination Disorder*" OR "Motor Skills Disorder*" OR "childhood dyspraxia" OR "developmental disorder of motor function*" OR "clumsy child syndrome*" OR "motor development disorder*" OR "Stereotypic Movement Disorder*" OR "Body Rocking" OR "Body-Focused Repetitive Behavio*" OR "Head Banging" OR trichotillomania OR "Tic Disorder*" OR "Tourette syndrome*" OR "Gilles de la Tourette*")

AND

(child* OR schoolchild* OR infan* OR adolescen* OR pediatri* OR paediatr* OR boy OR boys OR boyhood OR girl OR girls OR girlhood OR youth OR youths OR teen OR teens OR teenager* OR puberty OR preschool* OR toddler* OR juvenile* OR “high school” or kindergarden)

AND

Quality of life instrument OR well-being OR wellbeing OR QoL OR Quality adjusted life year OR health related quality of life OR QALY

Results 8/9/2017:110

Google Scholar

allintitle:quality of life AND (review OR meta-analysis OR systematic review) AND (child OR youth OR adolescents OR adolescent OR infants OR infant OR newborn OR baby OR teen) -animal

<https://scholar.google.nl/scholar?hl=nl&as_sdt=1%2C5&as_vis=1&q=allintitle%3Aquality+of+life+AND+%28review+OR+meta-analysis+OR+systematic+review%29+AND+%28child+OR+youth+OR+adolescents+OR+adolescent+OR+infants+OR+infant+OR+newborn+OR+baby+OR+teen%29+-animal&btnG>=

results 27/10/2017: 68

Google nederlands

<https://www.google.com/search?num=50&lr=lang_nl&as_qdr=all&tbs=lr%3Alang_1nl&ei=jxvzWeTAMIGLaufwjqAC&q=intitle%3A%28Child+OR+adolescent%29+AND+%28mental+health%29+AND+intitle%3A%28review+OR+meta-analysis%29+AND+%22quality+of+life%22+-animal+filetype%3Apdf&oq=intitle%3A%28Child+OR+adolescent%29+AND+%28mental+health%29+AND+intitle%3A%28review+OR+meta-analysis%29+AND+%22quality+of+life%22+-animal+filetype%3Apdf&gs_l=psy-ab.3...14035.15345.0.15770.8.7.0.0.0.0.0.0..0.0....0...1.1.64.psy-ab..8.0.0....0.cwpaXyULXu0>

intitle:(Child OR adolescent) AND (mental health) AND intitle:(review OR meta-analysis) AND "quality of life" -animal filetype:pdf

Cosmin:

Search with under 18, HR Qol, self rated qol, mental, behavioral disorders>> 0 results

Search with under 18, HR Qol, mental, behavioral disorders>> 13 results

# Appendix 2. Search terms psychometric quality

**Pubmed**

| #1psychometry | (instrumentation[sh] OR Validation Studies[pt] OR "reproducibility of results"[MeSH Terms] OR reproducib*[tiab] OR "psychometrics" OR psychometr*[tiab] OR clinimetr*[tiab] OR clinometr*[tiab] OR  "observer variation" OR "observer variation"[Title/Abstract] OR "discriminant analysis") AND reliab*[tiab] OR unreliab*[tiab] OR valid*[tiab] OR coefficient[tiab] OR homogeneity[tiab] OR homogeneous[tiab] OR "internal consistency"[Title/Abstract] OR (cronbach*[tiab] AND (alpha[tiab] OR alphas[tiab])) OR (item[tiab] AND (correlation*[tiab] OR selection*[tiab] OR reduction*[tiab])) OR agreement[tiab] OR precision[tiab] OR imprecision[tiab] OR "precise values"[Title/Abstract] OR test-retest[tiab] OR (test[tiab] AND retest[tiab]) OR (reliab*[tiab] AND (test[tiab] OR retest[tiab])) OR stability[tiab] OR interrater[tiab] OR inter-rater[tiab] OR intrarater[tiab] OR intra-rater[tiab] OR intertester[tiab] OR inter-tester[tiab] OR intratester[tiab] OR intra-tester[tiab] OR interobserver[tiab] OR inter-observer[tiab] OR intraobserver[tiab] OR intra-observer[tiab] OR intertechnician[tiab] OR inter-technician[tiab] OR intratechnician[tiab] OR intra-technician[tiab] OR interexaminer[tiab] OR inter-examiner[tiab] OR intraexaminer[tiab] OR intra-examiner[tiab] OR interassay[tiab] OR inter-assay[tiab] OR intraassay[tiab] OR intra-assay[tiab] OR interindividual[tiab] OR inter-individual[tiab] OR intraindividual[tiab] OR intra-individual[tiab] OR interparticipant[tiab] OR inter-participant[tiab] OR intraparticipant[tiab] OR intra-participant[tiab] OR kappa[tiab] OR kappa's[tiab] OR kappas[tiab] OR repeatab*[tiab] OR ((replicab*[tiab] OR repeated[tiab]) AND (measure[tiab] OR measures[tiab] OR findings[tiab] OR result[tiab] OR results[tiab] OR test[tiab] OR tests[tiab])) OR generaliza*[tiab] OR generalisa*[tiab] OR concordance[tiab] OR (intraclass[tiab] AND correlation*[tiab]) OR discriminative[tiab] OR "known group"[Title/Abstract] OR factor analysis[tiab] OR factor analyses[tiab] OR dimension*[tiab] OR subscale*[tiab] OR (multitrait[tiab] AND scaling[tiab] AND (analysis[tiab] OR analyses[tiab])) OR “item discriminant”[tiab] OR inter scale correlation*[tiab] OR error[tiab] OR errors[tiab] OR "individual variability"[Title/Abstract] OR (variability[tiab] AND (analysis[tiab] OR values[tiab])) OR (uncertainty[tiab] AND (measurement[tiab] OR measuring[tiab])) OR "standard error of measurement"[Title/Abstract] OR sensitiv*[tiab] OR responsive*[tiab] OR ((minimal[tiab] OR minimally[tiab] OR clinical[tiab] OR clinically[tiab]) AND (important[tiab] OR significant[tiab] OR detectable[tiab]) AND (change[tiab] OR difference[tiab])) OR (small*[tiab] AND (real[tiab] OR detectable[tiab]) AND (change[tiab] OR difference[tiab])) OR meaningful change[tiab] OR "ceiling effect"[Title/Abstract] OR “floor effect”[Title/Abstract] OR "item response model"[Title/Abstract] OR IRT[tiab] OR Rasch[tiab] OR "differential item functioning"[Title/Abstract] OR DIF[tiab] OR "computer adaptive testing"[Title/Abstract] OR “item bank”[tiab] OR "cross cultural equivalence"[Title/Abstract] |
| --- | --- |
| #2instruments | “questionnaires” OR “questionnaire” OR “self report” OR “self reports” OR “generic instruments” |
| #3 youth | (child*[tw] OR schoolchild*[tw] OR infan*[tw] OR adolescen*[tw] OR pediatri*[tw] OR paediatr*[tw] OR boy[tw] OR boys[tw] OR boyhood[tw] OR girl[tw] OR girls[tw] OR girlhood[tw] OR youth[tw] OR youths[tw] OR teen[tw] OR teens[tw] OR teenager*[tw] OR puberty[tw] OR preschool*[tw] OR toddler*[tw] OR juvenile*[tw] OR ‘high school’[tw] or kindergarten[tw]) |
| #4 excluded | (“addresses”[Publication Type] OR “biography”[Publication Type] OR “case reports”[Publication Type] OR “comment”[Publication Type] OR “directory”[Publication Type] OR “editorial”[Publication Type] OR “festschrift”[Publication Type] OR “interview”[Publication Type] OR “lectures”[Publication Type] OR “legal cases”[Publication Type] OR “legislation”[Publication Type] OR “letter”[Publication Type] OR “news”[Publication Type] OR “newspaper article”[Publication Type] OR “patient education handout”[Publication Type] OR “popular works”[Publication Type] OR “congresses”[Publication Type] OR “consensus development conference”[Publication Type] OR “consensus development conference, nih”[Publication Type] OR “practice guideline”[Publication Type]) NOT (“animals”[MeSH Terms] NOT “humans”[MeSH Terms]) |
|  | #1 AND #2 AND #3 NOT #4 |
| #5Specific list | 1= Chip OR “Child Health and illness profile”  2= CHQ OR “child health Questionnaire”  3= DUX-25 OR “Dutch-child-AZL-TNO-Quality-of-life”  4= KINDL OR KINDL-R OR “munich Quality of life questionnaire for children”  5= PEDSQL OR “Pediatric Quality of Life Inventory”  6= TACQOL OR “TNO AZL Child quality of life”  7= YQOL OR “Youth quality of life instrument”  8= HUI2 OR HUI3 OR “health utilities index”  9= AQOL OR “Adolescent Quality of life mental health scale”  10= EQ-5D OR “EuroQol five dimensions health questionnaire Youth”  11= ISLQ OR “inventory of subjective life quality for children and adolescents”  12= MSLSS OR “multidimensional student’s life satisfaction scale”  13= QOLPAV OR “quality of life profile adolescent version”  14= ITQOL OR infant and toddler Quality of life questionnaire  15= Lindstrom’s Quality of life model for children  16= Kidscreen  17=CHU9D OR child health utility index 9D  18= 16d OR “Sixteen dimensional measure of HRQOL”  19= 17d OR “seventeen dimensional measure of HRQOL”  20= CQOL OR “Child quality of life questionnaire”  21 = AHUM OR “adolescent health utility measure”  22= CHSCS OR “Comprehensive Health status classification system”  23 = CHQ OR “Generic children’s quality of life questionnaire”  24= QWB OR “Quality of well-being Scale”  25= TAPQOL OR (TNO AZL preschool quality of life) |
|  |  |

**Psycinfo**

| #1psychometry | instrumentation OR Validation Studies OR ‘reproducibility of results’ OR reproducib*OR ‘psychometrics’ OR psychometr* OR clinimetr* OR clinometr* reliab* OR unreliab* OR valid*OR ‘internal consistency’ OR item OR correlation* OR selection* OR reduction*) OR test-retest OR stability OR interrater OR inter-rater OR intrarater OR intra-rater OR intertester OR inter-tester OR intratester OR intra-tester OR interobserver OR inter-observer OR intraobserver OR intra-observer OR repeatab* replica* OR generaliza*OR generalisa*OR inter scale correlation*OR error OR errors OR ‘individual variability’ OR analysis OR values OR uncertainty measurement OR measuring OR ‘standard error of measurement’ OR sensitiv* OR ‘cross-cultural equivalence’ |
| --- | --- |
| #2instruments | ‘questionnaires’ OR ‘questionnaire’ OR ‘self report’ OR ‘self reports’ OR ‘generic instruments’ |
| #3 youth | TI (child* OR schoolchild* OR infan* OR adolescen* OR pediatri* OR paediatr* OR boy OR boys OR boyhood OR girl OR girls OR girlhood OR youth OR youths OR teen OR teens OR teenager* OR puberty OR preschool* OR toddler* OR juvenile* OR **“high school” or kindergarten**) OR AB (child* OR schoolchild* OR infan* OR adolescen* OR pediatri* OR paediatr* OR boy OR boys OR boyhood OR girl OR girls OR girlhood OR youth OR youths OR teen OR teens OR teenager* OR puberty OR preschool* OR toddler* OR juvenile* OR **“high school” or kindergarten**) OR AB (child* OR schoolchild* OR infan* OR adolescen* OR pediatri* OR paediatr* OR boy OR boys OR boyhood OR girl OR girls OR girlhood OR youth OR youths OR teen OR teens OR teenager* OR puberty OR preschool* OR toddler* OR juvenile* OR **“high school” or kindergarten**) OR AB (child* OR schoolchild* OR infan* OR adolescen* OR pediatri* OR paediatr* OR boy OR boys OR boyhood OR girl OR girls OR girlhood OR youth OR youths OR teen OR teens OR teenager* OR puberty OR preschool* OR toddler* OR juvenile* OR **“high school” or kindergarten**) |
|  | S1 AND S2 AND S3 |
| Specific list | 1= Chip OR “Child Health and illness profile”  2= CHQ OR “child health Questionnaire”  3= DUX-25 OR “Dutch-child-AZL-TNO-Quality-of-life”  4= KINDL OR KINDL-R OR “munich Quality of life questionnaire for children”  5=PEDSQL OR “Pediatric Quality of Life Inventory”  6= TACQOL OR “TNO AZL Child quality of life”  7= YQOL OR “Youth quality of life instrument”  8= HUI2 OR HUI3 OR “health utilities index”  9= AQOL OR “Adolescent Quality of life mental health scale”  10= EQ-5D OR “EuroQol five dimensions health questionnaire Youth”  11= ISLQ OR “inventory of subjective life quality for children and adolescents”  12= MSLSS OR “multidimensional student’s life satisfaction scale”  13= QOLPAV OR “quality of life profile adolescent version”  14= ITQOL OR infant and toddler Quality of life questionnaire  15= Lindstrom’s Quality of life model for children  16= Kidscreen  17=CHU9D OR child health utility index 9D  18= 16d OR “Sixteen dimensional measure of HRQOL”  19= 17d OR “seventeen dimensional measure of HRQOL”  20= CQOL OR “Child quality of life questionnaire”  21 = AHUM OR “adolescent health utility measure”  22= CHSCS OR “Comprehensive Health status classification system”  23 = CHQ OR “Generic children’s quality of life questionnaire”  24= QWB OR “Quality of well-being Scale”  25= TAPQOL OR (TNO AZL preschool quality of life) |

ECONLIT

(via ebscohost)

| #1psychometry | instrumentation OR Validation Studies OR ‘reproducibility of results’ OR reproducib*OR ‘psychometrics’ OR psychometr* OR clinimetr* OR clinometr* reliab* OR unreliab* OR valid*OR ‘internal consistency’ OR item OR correlation* OR selection* OR reduction*) OR test-retest OR stability OR interrater OR inter-rater OR intrarater OR intra-rater OR intertester OR inter-tester OR intratester OR intra-tester OR interobserver OR inter-observer OR intraobserver OR intra-observer OR repeatab* replica* OR generaliza*OR generalisa*OR inter scale correlation*OR error OR errors OR ‘individual variability’ OR analysis OR values OR uncertainty measurement OR measuring OR ‘standard error of measurement’ OR sensitiv* OR ‘cross-cultural equivalence’ |
| --- | --- |
| #2instruments | ‘questionnaires’ OR ‘questionnaire’ OR ‘self report’ OR ‘self reports’ OR ‘generic instruments’ |
| #3 youth | TI (child* OR schoolchild* OR infan* OR adolescen* OR pediatri* OR paediatr* OR boy OR boys OR boyhood OR girl OR girls OR girlhood OR youth OR youths OR teen OR teens OR teenager* OR puberty OR preschool* OR toddler* OR juvenile* OR **“high school” or kindergarten**) OR AB (child* OR schoolchild* OR infan* OR adolescen* OR pediatri* OR paediatr* OR boy OR boys OR boyhood OR girl OR girls OR girlhood OR youth OR youths OR teen OR teens OR teenager* OR puberty OR preschool* OR toddler* OR juvenile* OR **“high school” or kindergarten**) OR AB (child* OR schoolchild* OR infan* OR adolescen* OR pediatri* OR paediatr* OR boy OR boys OR boyhood OR girl OR girls OR girlhood OR youth OR youths OR teen OR teens OR teenager* OR puberty OR preschool* OR toddler* OR juvenile* OR **“high school” or kindergarten**) OR AB (child* OR schoolchild* OR infan* OR adolescen* OR pediatri* OR paediatr* OR boy OR boys OR boyhood OR girl OR girls OR girlhood OR youth OR youths OR teen OR teens OR teenager* OR puberty OR preschool* OR toddler* OR juvenile* OR **“high school” or kindergarten**) |
|  | S1 AND S2 AND S3 |
| Specific list | 1= Chip OR “Child Health and illness profile”  2= CHQ OR “child health Questionnaire”  3= DUX-25 OR “Dutch-child-AZL-TNO-Quality-of-life”  4= KINDL OR KINDL-R OR “munich Quality of life questionnaire for children”  5=PEDSQL OR “Pediatric Quality of Life Inventory”  6= TACQOL OR “TNO AZL Child quality of life”  7= YQOL OR “Youth quality of life instrument”  8= HUI2 OR HUI3 OR “health utilities index”  9= AQOL OR “Adolescent Quality of life mental health scale”  10= EQ-5D OR “EuroQol five dimensions health questionnaire Youth”  11= ISLQ OR “inventory of subjective life quality for children and adolescents”  12= MSLSS OR “multidimensional student’s life satisfaction scale”  13= QOLPAV OR “quality of life profile adolescent version”  14= ITQOL OR infant and toddler Quality of life questionnaire  15= Lindstrom’s Quality of life model for children  16= Kidscreen  17=CHU9D OR child health utility index 9D  18= 16d OR “Sixteen dimensional measure of HRQOL”  19= 17d OR “seventeen dimensional measure of HRQOL”  20= CQOL OR “Child quality of life questionnaire”  21 = AHUM OR “adolescent health utility measure”  22= CHSCS OR “Comprehensive Health status classification system”  23 = CHQ OR “Generic children’s quality of life questionnaire”  24= QWB OR “Quality of well-being Scale”  25= TAPQOL OR (TNO AZL preschool quality of life) |

Embase.com

| #1psychometry | 'intermethod comparison'/exp OR 'data collection method'/exp OR 'validation study'/exp OR 'feasibility study'/exp OR 'pilot study'/exp OR 'psychometry'/exp OR 'reproducibility'/exp OR reproducib*:ab,ti OR 'audit':ab,ti OR psychometr*:ab,ti OR clinimetr*:ab,ti OR clinometr*:ab,ti OR 'observer variation'/exp OR 'observer variation':ab,ti OR 'discriminant analysis'/exp OR 'validity'/exp OR reliab*:ab,ti OR valid*:ab,ti OR 'coefficient':ab,ti OR 'internal consistency':ab,ti OR (cronbach*:ab,ti AND ('alpha':ab,ti OR 'alphas':ab,ti)) OR 'item correlation':ab,ti OR 'item correlations':ab,ti OR 'item selection':ab,ti OR 'item selections':ab,ti OR 'item reduction':ab,ti OR 'item reductions':ab,ti OR 'agreement':ab,ti OR 'precision':ab,ti OR 'imprecision':ab,ti OR 'precise values':ab,ti OR 'test-retest':ab,ti OR ('test':ab,ti AND 'retest':ab,ti) OR (reliab*:ab,ti AND ('test':ab,ti OR 'retest':ab,ti)) OR 'stability':ab,ti OR 'interrater':ab,ti OR 'inter-rater':ab,ti OR 'intrarater':ab,ti OR 'intra-rater':ab,ti OR 'intertester':ab,ti OR 'inter-tester':ab,ti OR 'intratester':ab,ti OR 'intra-tester':ab,ti OR 'interobeserver':ab,ti OR 'inter-observer':ab,ti OR 'intraobserver':ab,ti OR 'intra-observer':ab,ti OR 'intertechnician':ab,ti OR 'inter-technician':ab,ti OR 'intratechnician':ab,ti OR 'intra-technician':ab,ti OR 'interexaminer':ab,ti OR 'inter-examiner':ab,ti OR 'intraexaminer':ab,ti OR 'intra-examiner':ab,ti OR 'interassay':ab,ti OR 'inter-assay':ab,ti OR 'intraassay':ab,ti OR 'intra-assay':ab,ti OR 'interindividual':ab,ti OR 'inter-individual':ab,ti OR 'intraindividual':ab,ti OR 'intra-individual':ab,ti OR 'interparticipant':ab,ti OR 'inter-participant':ab,ti OR 'intraparticipant':ab,ti OR 'intra-participant':ab,ti OR 'kappa':ab,ti OR 'kappas':ab,ti OR 'coefficient of variation':ab,ti OR repeatab*:ab,ti OR ((replicab*:ab,ti OR 'repeated':ab,ti) AND ('measure':ab,ti OR 'measures':ab,ti OR 'findings':ab,ti OR 'result':ab,ti OR 'results':ab,ti OR 'test':ab,ti OR 'tests':ab,ti)) OR generaliza*:ab,ti OR generalisa*:ab,ti OR 'concordance':ab,ti OR ('intraclass':ab,ti AND correlation*:ab,ti) OR 'discriminative':ab,ti OR 'known group':ab,ti OR 'factor analysis':ab,ti OR 'factor analyses':ab,ti OR 'factor structure':ab,ti OR 'factor structures':ab,ti OR 'dimensionality':ab,ti OR subscale*:ab,ti OR 'multitrait scaling analysis':ab,ti OR 'multitrait scaling analyses':ab,ti OR 'item discriminant':ab,ti OR 'interscale correlation':ab,ti OR 'interscale correlations':ab,ti OR (('error':ab,ti OR 'errors':ab,ti) AND (measure*:ab,ti OR correlat*:ab,ti OR evaluat*:ab,ti OR 'accuracy':ab,ti OR 'accurate':ab,ti OR 'precision':ab,ti OR 'mean':ab,ti)) OR 'individual variability':ab,ti OR 'interval variability':ab,ti OR 'rate variability':ab,ti OR 'variability analysis':ab,ti OR ('uncertainty':ab,ti AND ('measurement':ab,ti OR 'measuring':ab,ti)) OR 'standard error of measurement':ab,ti OR sensitiv*:ab,ti OR responsive*:ab,ti OR ('limit':ab,ti AND 'detection':ab,ti) OR 'minimal detectable concentration':ab,ti OR interpretab*:ab,ti OR (small*:ab,ti AND ('real':ab,ti OR 'detectable':ab,ti) AND ('change':ab,ti OR 'difference':ab,ti)) OR 'meaningful change':ab,ti OR 'minimal important change':ab,ti OR 'minimal important difference':ab,ti OR 'minimally important change':ab,ti OR 'minimally important difference':ab,ti OR 'minimal detectable change':ab,ti OR 'minimal detectable difference':ab,ti OR 'minimally detectable change':ab,ti OR 'minimally detectable difference':ab,ti OR 'minimal real change':ab,ti OR 'minimal real difference':ab,ti OR 'minimally real change':ab,ti OR 'minimally real difference':ab,ti OR 'ceiling effect':ab,ti OR 'floor effect':ab,ti OR 'item response model':ab,ti OR 'irt':ab,ti OR 'rasch':ab,ti OR 'differential item functioning':ab,ti OR 'dif':ab,ti OR 'computer adaptive testing':ab,ti OR 'item bank':ab,ti OR 'cross-cultural equivalence':ab,ti |
| --- | --- |
| #2instruments | ‘questionnaires’ OR ‘questionnaire’ OR ‘self report’ OR ‘self reports’ OR ‘generic instruments’ |
| #3 child | **(child***:ab,ti OR **schoolchild***:ab,ti OR **infan***:ab,ti OR **adolescen***:ab,ti OR **pediatri***:ab,ti OR **paediatr***:ab,ti OR **boy**:ab,ti OR **boys**:ab,ti OR **boyhood**:ab,ti OR **girl**:ab,ti OR **girls**:ab,ti OR **girlhood**:ab,ti OR **youth**:ab,ti OR **youths**:ab,ti OR **teen**:ab,ti OR **teens**:ab,ti OR **teenager***:ab,ti OR **puberty**:ab,ti OR **preschool***:ab,ti OR **toddler***:ab,ti OR **juvenile***:ab,ti) |
|  | #1 AND #2 AND #3 |
| Specific list | 1= Chip OR ‘Child Health and illness profile’  2= CHQ OR ‘child health Questionnaire’  3= DUX-25 OR ‘Dutch child AZL TNO Quality of life’  4= KINDL OR KINDL-R OR ‘munich Quality of life questionnaire for children’  5=PEDSQL OR ‘Pediatric Quality of Life Inventory’  6= TACQOL OR ‘TNO AZL Child quality of life’  7= YQOL OR ‘Youth quality of life instrument’  8= HUI2 OR HUI3 OR ‘health utilities index’  9= AQOL OR ‘Adolescent Quality of life mental health scale’  10= EQ-5D OR ‘EuroQol five dimensions health questionnaire Youth’  11= ISLQ OR ‘inventory of subjective life quality for children and adolescents’  12= MSLSS OR ‘multidimensional student’s life satisfaction scale’  13= QOLPAV OR ‘quality of life profile adolescent version’  14= ITQOL OR ‘infant and toddler Quality of life questionnaire’  15= Lindstrom’s Quality of life model for children  16= Kidscreen  17=CHU9D OR ‘child health utility index 9D’  18= 16d OR ‘Sixteen dimensional measure of HRQOL’  19= 17d OR ‘seventeen dimensional measure of HRQOL’  20= CQOL OR ‘Child quality of life questionnaire’  21 = AHUM OR ‘adolescent health utility measure’  22= CHSCS OR ‘Comprehensive Health status classification system’  23 = CHQ OR ‘Generic children’s quality of life questionnaire’  24= QWB OR ‘Quality of well-being Scale’  25= TAPQOL OR (TNO AZL preschool quality of life) |

# Appendix 3. Cosmin Definitions

**COSMIN checklist**

| **Cosmin concept** | **Cosmin definition** | **Additional explanation/definition** | **The statistical methods according to the COSMIN checklist** |
| --- | --- | --- | --- |
| **Internal consistency** | The degree of the interrelatedness among the items | The degree of consistency of measuring the same construct across items within a test or subscale. This is based on the correlations between different items of an instrument or the same subscale. | Classical Test Theory (CTT), continuous scores: Cronbach’s alpha calculated  CTT, dichotomous scores: Cronbach’s alpha or KR-20 calculated  IRT: a goodness of fit statistic at a global level calculated e.g. χ2, reliability coefficient of estimated latent trait value (index of (subject or item) separation |
| **Reliability** | The proportion of the total variance in the measurements which is due to ‘true’ differences between patients. The word ‘true’ must be seen in the context of the CTT, which states that any observation is composed of two components – a true score and error associated with the observation. ‘True’ is the average score that would be obtained if the scale were given an infinite number of times. It refers only to the consistency of the score, and not to its accuracy (ref Streiner & Norman). | the degree of agreement between the repeated measurements.  Consists of different forms of reliability:   1. Test-retest-reliability 2. inter-rater reliability 3. intra-rater reliability | continuous scores: ICC  dichotomous/nominal/ordinal scores: kappa  ordinal scores: weighted kappa |
| **Measurement error** | The systematic and random error of a patient’s score that is not attributed to true changes in the construct to be measured. |  | CTT: Standard Error of Measurement (SEM), Smallest Detectable Change (SDC) or Limits of Agreement (LoA) |
| **Content validitity** | The degree to which the content of an HR-PRO instrument is an adequate reflection of the construct to be measured. | Was there an assessment of whether all items refer to relevant aspects of the construct, for the study population, the purpose of the instrument to be measured? Also was there an assessment of whether all items together comprehensively reflect the construct to be measured?  Includes face-validity |  |
| **Structural validitity** | The degree to which the scores of an HR-PRO instrument are an adequate reflection of the dimensionality of the construct to be measured. | Does the scale consist of effect indicators, i.e. is it based on a reflective model? | CTT: exploratory or confirmatory factor analysis  IRT: |
| **Hypotheses testing** | The degree to which the scores of an HR-PRO instrument are consistent with hypotheses (for instance with regard to internal relationships, relationships to scores of other instruments, or differences between relevant groups) based on the assumption that the HR-PRO instrument validly measures the construct to be measured. | Construct validity: the extent to which operationalizations of a construct measure a construct as defined by a theory.  Includes:   - Construct validity^[[1]](#footnote-1)^ - Convergent validity or Discriminant validity - Divergent validity | Were design and statistical methods adequate for the hypotheses to be tested? |
| **Cross-cultural validity**^[[2]](#footnote-2)^ | The degree to which the performance of the items on a translated or culturally adapted HR-PRO instrument are an adequate reflection of the performance of the items of the original version of the HR-PRO instrument. |  | CTT: confirmatory factor analysis  IRT: differential item function (DIF) between language groups |
| **Criterion validity** | The degree to which the scores of an HR-PRO instrument are an adequate reflection of a ‘gold standard’. | the correlation between the test and a criterion variable (or variables) taken as representative of the construct. it compares the test with other measures or outcomes.  If the test data and criterion data are collected at the same time, this is referred to as concurrent validity evidence. Examples of concurrent validity: Degree of (dis)agreement between parent/children or father/mother.  Or degree of agreement between self-report data and administrative data.  If the test data are collected first in order to predict criterion data collected at a later point in time, then this is referred to as predictive validity evidence. | continuous scores: correlations, or the area under the receiver operating curve calculated  dichotomous scores: sensitivity and specificity determined |
| **Responsiveness** | The ability of an HR-PRO instrument to detect change over time in the construct to be measured. |  |  |
| **Feasibility** | Seems the (new) measure feasible and practible? | **Acceptability:** To what extent is a new idea, program, process or measure judged as suitable, satisfying, or attractive to program deliverers? To program recipients?  **Demand**: To what extent is a new idea, program, process, or measure likely to be used (i.e., how much demand is likely to exist?)  **Implementation**: To what extent can a new idea, program, process, or measure be successfully delivered to intended participants in some defined, but not fully controlled, context?  **Practicality** To what extent can an idea, program, process, or measure be carried out with intended participants using existing means, resources, and circumstances and without outside intervention?  **Adaptation** To what extent does an existing idea, program, process, or measure perform when changes are made for a new format or with a different population?  **Integration** To what extent can a new idea, program, process, or measure be integrated within an existing system?  **Expansion** To what extent can a previously tested program, process, approach, or system be expanded to provide a new program or service?  **Limited efficacy** Does the a new idea, program, process, or measure show promise of being successful with the intended population, even in a highly controlled setting?^[[3]](#footnote-3)^ |  |

# Appendix 4. Quality scores Questionnaires.

| Instrument | Domains relevant for mental health | | Number of psychometric studies in general population | | Number of psychometric studies in youth with mental health problems | | Psychometric quality in youth with mental health problems | | Valueset | | Total score |
| --- | --- | --- | --- | --- | --- | --- | --- | --- | --- | --- | --- |
| CHIP | Satisfaction, comfort, risk avoidance, resilience, achievement | 2 | 12 | 2 | 3 | 1 | Medium to good | 1 | None | 0 | 6 |
| CHQ | role limitations-emotional/behavioral, behavior, mental health, self-esteem, parental impact–emotional, family activities, family cohesion | 2 | 33 | 2 | 2 | 1 | Medium to good | 1 | None | 0 | 6 |
| KINDL-R | self-esteem, family, social contacts, school | 2 | 19 | 2 | 2 | 1 | Low quality other study mixed results | 0 | None | 0 | 5 |
| PedsQL | school functioning, emotional functioning, social functioning | 1 | 50 | 2 | 5 | 2 | Low quality in young children good quality in older children: Mixed results | 1 | None | 0 | 6 |
| TACQOL | social functioning (social), cognitive functioning (cognition), positive psychological functioning (emopos), negative psychological functioning (emoneg) | 2 | 3 | 0 | 0 | 0 | None performed | 0 | None | 0 | 2 |
| TAPQOL | social functioning: play with peers, self-esteem, social comfort, problem behavior; cognitive functioning: understanding what others say, speech, elaborating in expressive language; emotional functioning: mood, anxiety and liveliness | 2 | 7 | 1 | 1 | 0 | Medium scores | 1 | None | 0 | 4 |
| YQOL | Sense of self, social relationships, culture and community, general quality of life | 2 | 3 | 0 | 2 | 1 | Good quality | 2 | None | 0 | 5 |
| HUI | emotion, cognition, | 1 | 1 | 0 | 0 | 0 | None performed | 0 | Adult value set | 1 | 2 |
| AQOL-MS | self, peers, family, school, environment | 2 | 2 | 0 | 2 | 1 | Good quality | 1 | None | 0 | 4 |
| AQOL 6D | social and family relationships, mental health, | 1 | 2 | 0 | 0 | 0 | None performed | 0 | Adult value set | 1 | 2 |
| EQ-5d-Y | feeling worried, sad or unhappy | 0 | 15 | 2 | 6 | 2 | Mixed results | 1 | Adult value set | 1 | 6 |
| MSLSS | family, friends, school, living environment, self | 2 | 8 | 1 | 1 | 0 | Mixed results | 1 | None | 0 | 4 |
| QOLPAV | being (psychological, spiritual), beloning (social, community), becoming (practical, leisure, growth) | 2 | 5 | 1 | 0 | 0 | None performed | 0 | None | 0 | 3 |
| ITQOL | infant concepts: temperament and moods, general behavior perceptions, getting along with others, parent concepts: impact-emotional, impact-time, mental health, , family cohesion | 2 | 2 | 0 | 0 | 0 | None performed | 0 | None | 0 | 2 |
| KIDSCREEN | psychological well-being, moods and emotions, self-perception, autonomy, parent relations and home life, social support and peers, school environment, social acceptance (bullying) | 2 | 34 | 2 | 3 | 1 | Mixed results | 1 | None | 0 | 6 |
| CHU9D | worried, sad, annoyed, school work/homework, sleep | 2 | 7 | 1 | 2 | 1 | Good | 2 | Adult value set | 1 | 7 |
| 16D | sleeping, eating, speech, school and hobbies, learning and memory, depression, distress, vitality, appearance, friends, concentration | 2 | 2 | 0 | 0 | 0 | None performed | 0 | Child value set | 2 | 4 |
| 17D | sleeping, eating, speech, school and hobbies, learning and memory, depression, distress, vitality, appearance, friends, concentration | 2 | 1 | 0 | 0 | 0 | None performed | 0 | Child value set | 2 | 4 |
| CQOL | school, out of school activities, friends, family relationships, worries, depression, communication, eating, sleep, appearance | 2 | 1 | 0 | 1 | 0 | Mixed results | 1 | None | 0 | 3 |
| AHUM | self-image, health perceptions | 1 | 0 | 0 | 0 | 0 | 0 | 0 | Adult value set | 1 | 2 |
| CHSCS | emotion, learn/remember, think/problem-solve, behavior | 2 | 1 | 0 | 0 | 0 | None performed | 0 | None | 0 | 2 |
| GCQ |  | 0 | 1 | 0 | 0 | 0 | None performed | 0 | None | 0 | 0 |
| QWB | social activity including the role of expectations | 0 | 1 | 0 | 1 | 0 | Good quality | 2 | Adult value set | 1 | 3 |

Domains relevant for mental health.

0: 1 or less

1: 1-3

2: 4 or more

Number of psychometric studies:

0: 3 or less studies

1: 4-9 studies

2: 10 or more

Number of psychometric studies:

0: 1 or less studies

1: 2-5 studies

2: 5 or more

Psychometric quality

0: low quality

1: medium quality or mixed results

2: good or excellent quality

Value set:

0: no value set

1: valueset for adults

2: valueset for youth

max total: 10

# Appendix 5. PRISMA flow charts Review of reviews


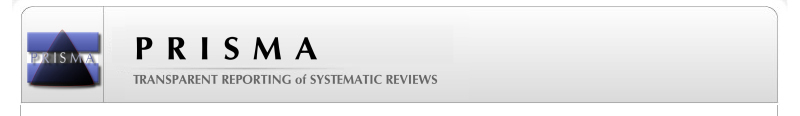


Full-text articles excluded, with reasons
(Wrong publication type n = 6

No instruments described n = 4

No HRQoL instruments n = 3

Not suitable for use in psychosocial problems n = 1 )

Records excluded
(n = 1218 )

Records screened
(n = 1261)

Records after duplicates removed
(n = 1261 )

Identification

Eligibility

Included

Screening

Studies included in qualitative synthesis
(n = 29)

Additional records identified through other sources
(n = 2 literature lists

n=6 Cosmin database

n=5 Google Scholar

n=2 Picarta )

Full-text articles assessed for eligibility
(n = 43)

Records identified through database searching
(n = 1636 )

# Appendix 6. Prisma Flow chart Psychometric characteristics


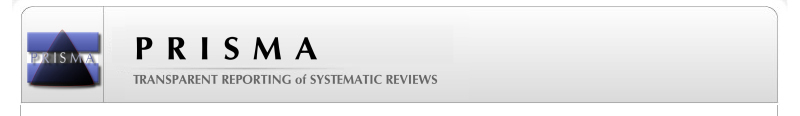
**PRISMA 2009 Flow Diagram- 16D**

Studies included in qualitative synthesis
(n = 2)

Full-text articles assessed for eligibility
(n = 2)

Records excluded
(n = 15)

Records screened
(n = 17)

Records after duplicates removed
(n = 17)

Additional records identified through other sources
(n = 0)

Identification

Eligibility

Included

Screening

Records identified through database searching
(n = 27)

Full-text articles excluded, with reasons
(n = 0)


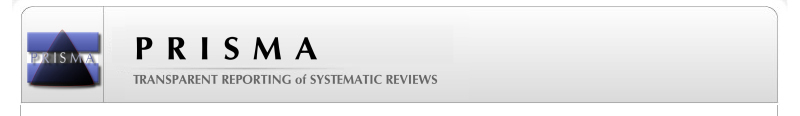
**PRISMA 2009 Flow Diagram- 17D**

Studies included in qualitative synthesis
(n = 1)

Full-text articles excluded, with reasons
(n = 0)

Full-text articles assessed for eligibility
(n = 1)

Records excluded
(n = 15)

Records screened
(n = 16)

Records after duplicates removed
(n = 16)

Additional records identified through other sources
(n = 0)

Identification

Eligibility

Included

Screening

Records identified through database searching
(n = 20)


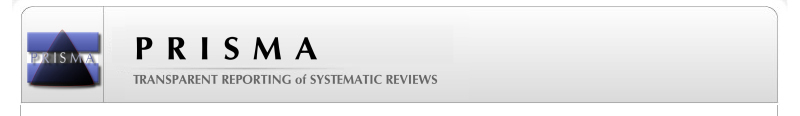
**PRISMA 2009 Flow Diagram-AHUM**

Studies included in qualitative synthesis
(n = 0)

Full-text articles assessed for eligibility
(n = 1)

Records excluded
(n = 3)

Records screened
(n = 4)

Records after duplicates removed
(n = 4)

Additional records identified through other sources
(n = 0)

Identification

Eligibility

Included

Screening

Records identified through database searching
(n = 5)

Full-text articles excluded, with reasons
(n = 1)

No psychometric research


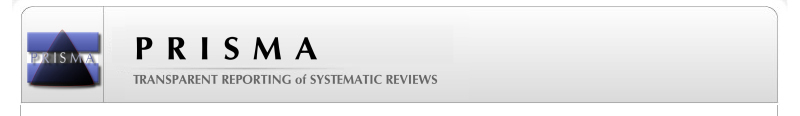
**PRISMA 2009 Flow Diagram-AQOL**

Studies included in qualitative synthesis
(n = 6)

Full-text articles assessed for eligibility
(n = 6)

Records excluded
(n = 398)

Records screened
(n = 404)

Records after duplicates removed
(n = 404)

Additional records identified through other sources
(n = 0)

Identification

Eligibility

Included

Screening

Records identified through database searching
(n = 412)

Full-text articles excluded, with reasons
(n = 0)


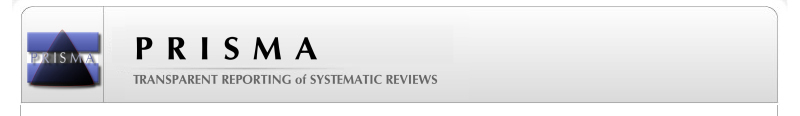
**PRISMA 2009 Flow Diagram-CHIP**

Studies included in qualitative synthesis
(n = 12)

Full-text articles assessed for eligibility
(n = 13)

Records excluded
(n = 361)

Records screened
(n = 374)

Records after duplicates removed
(n = 374)

Additional records identified through other sources
(n = 0)

Identification

Eligibility

Included

Screening

Records identified through database searching
(n = 478)

Full-text articles excluded, with reasons
(n = 1)

Duplicate


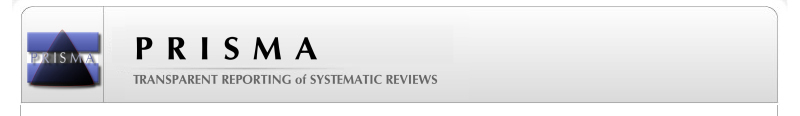
**PRISMA 2009 Flow Diagram-CHQ**

Studies included in qualitative synthesis
(n = 33)

Full-text articles assessed for eligibility
(n = 39)

Records excluded
(n = 974)

Records screened
(n = 1013)

Records after duplicates removed
(n = 1013)

Additional records identified through other sources
(n = 0)

Identification

Eligibility

Included

Screening

Records identified through database searching
(n = 1499)

Full-text articles excluded, with reasons
(n = 6)

Wrong language (n = 1)

Wrong Sample (n = 3)

Review (n = 1)

No psychometric research (n = 1)


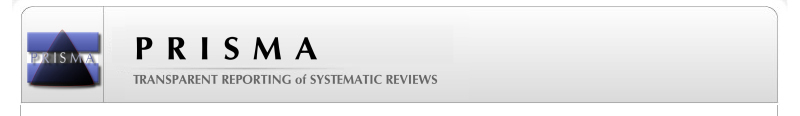
**PRISMA 2009 Flow Diagram-CHSCS**

Studies included in qualitative synthesis
(n = 3)

Full-text articles assessed for eligibility
(n = 3)

Records excluded
(n = 33)

Records screened
(n = 36)

Records after duplicates removed
(n = 36)

Additional records identified through other sources
(n = 0)

Identification

Eligibility

Included

Screening

Records identified through database searching
(n = 40)

Full-text articles excluded, with reasons
(n = 0)


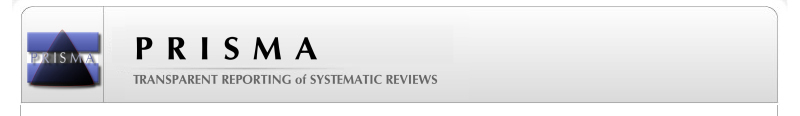
**PRISMA 2009 Flow Diagram-CHUD9**

Studies included in qualitative synthesis
(n = 7)

Full-text articles assessed for eligibility
(n = 11)

Records excluded
(n = 11)

Records screened
(n = 22)

Records after duplicates removed
(n = 22)

Additional records identified through other sources
(n = 0)

Identification

Eligibility

Included

Screening

Records identified through database searching
(n = 38)

Full-text articles excluded, with reasons
(n = 4)

No psychometric research


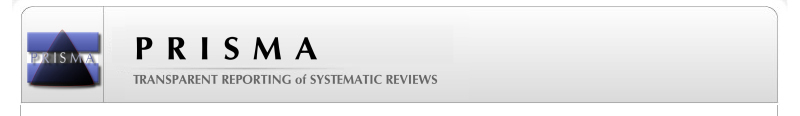
**PRISMA 2009 Flow Diagram-CQOL**

Studies included in qualitative synthesis
(n = 1)

Full-text articles excluded, with reasons
(n = 0)

Full-text articles assessed for eligibility
(n = 1)

Records excluded
(n = 15)

Records screened
(n = 16)

Records after duplicates removed
(n = 16)

Additional records identified through other sources
(n = 0)

Identification

Eligibility

Included

Screening

Records identified through database searching
(n = 20)


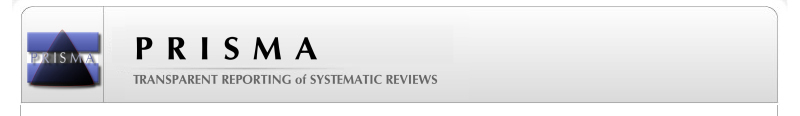
**PRISMA 2009 Flow Diagram-DUX-25**

Studies included in qualitative synthesis
(n = 0)

Full-text articles excluded, with reasons
(n = 0)

Full-text articles assessed for eligibility
(n = 0)

Records excluded
(n = 489)

Records screened
(n = 489)

Records after duplicates removed
(n = 489)

Additional records identified through other sources
(n = 0)

Identification

Eligibility

Included

Screening

Records identified through database searching
(n = 524)


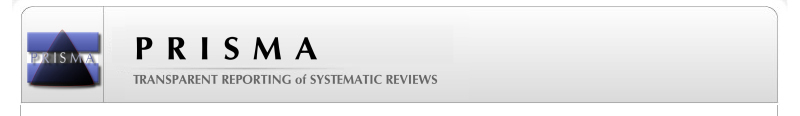
**PRISMA 2009 Flow Diagram-EQ-5D**

Studies included in qualitative synthesis
(n = 15)

Full-text articles assessed for eligibility
(n = 20)

Records excluded
(n = 20)

Records screened
(n = 515)

Records after duplicates removed
(n = 515)

Additional records identified through other sources
(n = 1)

Identification

Eligibility

Included

Screening

Records identified through database searching
(n = 635)

Full-text articles excluded, with reasons
(n = 5)

Review (n = 1)

No psychometric research (n = 4)


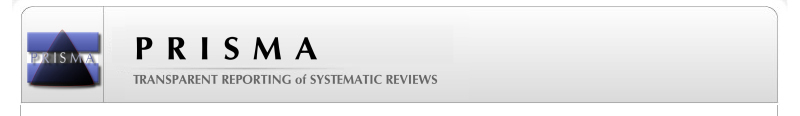
**PRISMA 2009 Flow Diagram-GCQ**

Studies included in qualitative synthesis
(n = 1)

Full-text articles assessed for eligibility
(n = 1)

Records excluded
(n = 179)

Records screened
(n = 180)

Records after duplicates removed
(n = 180)

Additional records identified through other sources
(n = 0)

Identification

Eligibility

Included

Screening

Records identified through database searching
(n = 187)

Full-text articles excluded, with reasons
(n = 0)


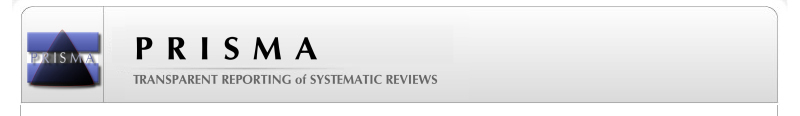
**PRISMA 2009 Flow Diagram-HUI2/3**

Studies included in qualitative synthesis
(n = 1)

Full-text articles assessed for eligibility
(n = 14)

Records excluded
(n = 931)

Records screened
(n = 945)

Records after duplicates removed
(n = 945)

Additional records identified through other sources
(n = 0)

Identification

Eligibility

Included

Screening

Records identified through database searching
(n = 1132)

Full-text articles excluded, with reasons
(n = 13)

Wrong language (n = 1)

Wrong sample (n = 8)

No psychometric research (n = 4)


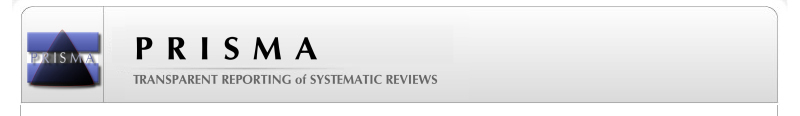
**PRISMA 2009 Flow Diagram-ITQOL**

Studies included in qualitative synthesis
(n = 3)

Full-text articles assessed for eligibility
(n = 3)

Records excluded
(n = 29)

Records screened
(n = 32)

Records after duplicates removed
(n = 32)

Additional records identified through other sources
(n = 0)

Identification

Eligibility

Included

Screening

Records identified through database searching
(n = 48)

Full-text articles excluded, with reasons
(n = 0)


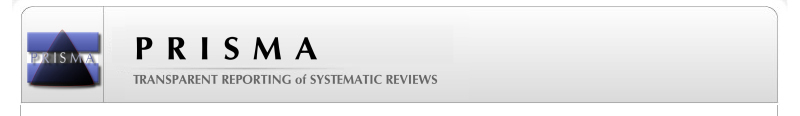
**PRISMA 2009 Flow Diagram-Kidscreen**

Studies included in qualitative synthesis
(n = 33)

Full-text articles assessed for eligibility
(n = 47)

Records excluded
(n = 385)

Records screened
(n = 432)

Records after duplicates removed
(n = 432)

Additional records identified through other sources
(n = 3)

Identification

Eligibility

Included

Screening

Records identified through database searching
(n = 663)

Full-text articles excluded, with reasons
(n = 12)

Wrong language (n = 4)

Wrong Sample (n = 1)

Wrong questionnaire (n = 1)

Review (n = 2)

No psychometric research (n = 2)

Congress abstract (n = 2)


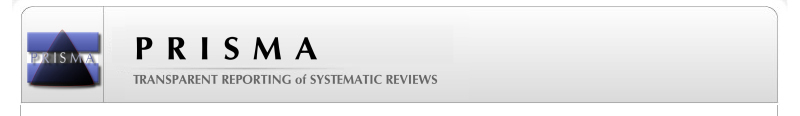
**PRISMA 2009 Flow Diagram-KINDL-R**

Studies included in qualitative synthesis
(n = 19)

Full-text articles assessed for eligibility
(n = 25)

Records excluded
(n = 321)

Records screened
(n = 346)

Records after duplicates removed
(n = 346)

Additional records identified through other sources
(n = 0)

Identification

Eligibility

Included

Screening

Records identified through database searching
(n = 499)

Full-text articles excluded, with reasons
(n = 6)

Wrong language (n = 4)

Wrong Sample (n = 1)

Duplicate (n = 1)


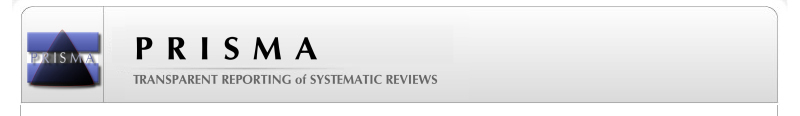
**PRISMA 2009 Flow Diagram-MSLSS**

Studies included in qualitative synthesis
(n = 8)

Full-text articles assessed for eligibility
(n = 9)

Records excluded
(n = 30)

Records screened
(n = 39)

Records after duplicates removed
(n = 39)

Additional records identified through other sources
(n = 0)

Identification

Eligibility

Included

Screening

Records identified through database searching
(n = 44)

Full-text articles excluded, with reasons
(n = 1)

Wrong sample


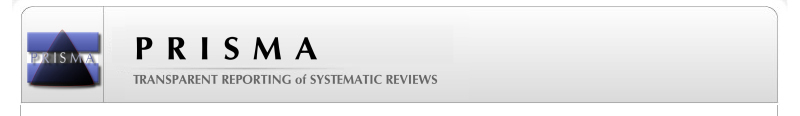
**PRISMA 2009 Flow Diagram-PedsQL**

Studies included in qualitative synthesis
(n = 50)

Full-text articles assessed for eligibility
(n = 57)

Records excluded
(n = 2595)

Records screened
(n = 2652)

Records after duplicates removed
(n = 2652)

Additional records identified through other sources
(n = 0)

Identification

Eligibility

Included

Screening

Records identified through database searching
(n = 2947)

Full-text articles excluded, with reasons
(n = 7)

Wrong language (n = 2)

Wrong Sample (n = 2)

Review (n = 2)

Duplicate (n = 1)


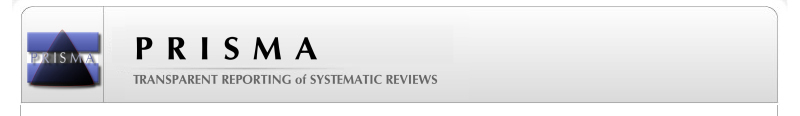
**PRISMA 2009 Flow Diagram-QOLPAV**

Studies included in qualitative synthesis
(n = 4)

Full-text articles assessed for eligibility
(n = 4)

Records excluded
(n = 5)

Records screened
(n = 9)

Records after duplicates removed
(n = 9)

Additional records identified through other sources
(n = 0)

Identification

Eligibility

Included

Screening

Records identified through database searching
(n = 17)

Full-text articles excluded, with reasons
(n = 0)


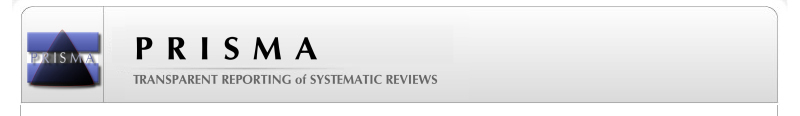
**PRISMA 2009 Flow Diagram-QWB**

Studies included in qualitative synthesis
(n = 2)

Full-text articles assessed for eligibility
(n = 2)

Records excluded
(n = 37)

Records screened
(n = 39)

Records after duplicates removed
(n = 39)

Additional records identified through other sources
(n = 0)

Identification

Eligibility

Included

Screening

Records identified through database searching
(n = 44)

Full-text articles excluded, with reasons
(n = 0)


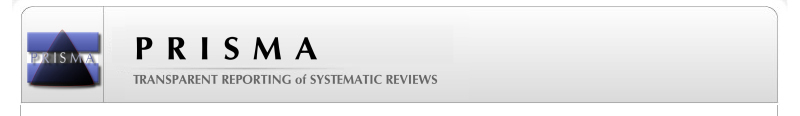
**PRISMA 2009 Flow Diagram-TACQOL**

Studies included in qualitative synthesis
(n = 3)

Full-text articles assessed for eligibility
(n = 3)

Records excluded
(n = 48)

Records screened
(n = 51)

Records after duplicates removed
(n = 51)

Additional records identified through other sources
(n = 1)

Identification

Eligibility

Included

Screening

Records identified through database searching
(n = 78)

Full-text articles excluded, with reasons
(n = 0)


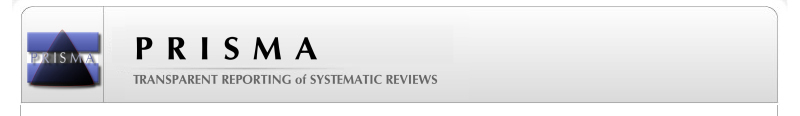
**PRISMA 2009 Flow Diagram-TAPQOL**

Studies included in qualitative synthesis
(n = 7)

Full-text articles assessed for eligibility
(n = 9)

Records excluded
(n = 43)

Records screened
(n = 52)

Records after duplicates removed
(n = 52)

Additional records identified through other sources
(n = 0)

Identification

Eligibility

Included

Screening

Records identified through database searching
(n = 88)

Full-text articles excluded, with reasons
(n = 2)

Wrong language (n = 1)

Duplicate (n = 1)


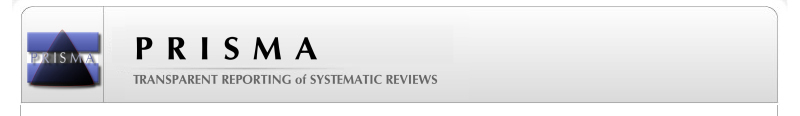
**PRISMA 2009 Flow Diagram-YQOL**

Studies included in qualitative synthesis
(n = 3)

Full-text articles assessed for eligibility
(n = 5)

Records excluded
(n = 1353)

Records screened
(n = 1358)

Records after duplicates removed
(n = 1358)

Additional records identified through other sources
(n = 0)

Identification

Eligibility

Included

Screening

Records identified through database searching
(n = 1422)

Full-text articles excluded, with reasons
(n = 3)

Wrong language

# Appendix 7 Summary Tables of psychometric research

### Table 1. 16D

| **Reference and instrument identification** | **Internal consistency** | **Reliability** | **Measurement error** | **Content validity** | **Structural validity** | **Hypotheses testing** | **Cross-cultural validity** | **Criterion validity** | **Responsive-ness** | **feasibility** |
| --- | --- | --- | --- | --- | --- | --- | --- | --- | --- | --- |
| **Apajasalo et al. (1996)** | **no** | **yes, repeatability coefficient 91%** | **no** | **no** | **no** | **yes, difference between girls and boys, proxy and self, and known groups of patients waiting for organ transplantation and controls (apajasalo (1996)** | **no** | **no** | **no** |  |
| **Grano et al. (2016)** | **yes (cronbachs alpha of .827) in the 11 to 15 year group** |  |  |  | **yes, exploratory factor analysis** | **yes, correlation with functioning ability (as measured with GAF scale ) of -.195 in the 11 to 15 year old group** |  |  |  |  |

Note. Developed by Apajasalo et al. Quality of Life Research (1996)

### Table 2. 17d

| **Reference and instrument identification** | **Internal consistency** | **Reliability** | **Measurement error** | **Content validity** | **Structural validity** | **Hypotheses testing** | **Cross-cultural validity** | **Criterion validity** | **Responsive-ness** | **feasibility** |
| --- | --- | --- | --- | --- | --- | --- | --- | --- | --- | --- |
| Apajasalo et al. 1996 | test-retest: 95% (only 2 dimensions below 92%, |  | yes expert panel p533 |  | construct: discriminatory power Between girls and boys, and patients and controls |  |  |  |  |  |

Note. Developed by Apajasalo et al. 1996

### Table 3. AQOL

| **Version** | **Reference and instrument identification** | **Internal consistency** | **Reliability** | **Measurement error** | **Content validity** | **Structural validity** | **Hypotheses testing** | **Cross-cultural validity** | **Criterion validity** | **Responsive-ness** | **feasibility** |
| --- | --- | --- | --- | --- | --- | --- | --- | --- | --- | --- | --- |
|  |  |  |  |  |  |  |  |  |  |  |  |
| **Assessment of Quality of Life 6D for adolescents (AQOL-6D)** | **Richardson et al (2012)** |  |  |  | **yes, Raykov's composite scale reliability: 0,054 (threshold: max. 0,08), Comparative Fit Index 0,97 (Richardson et al. p.4)** |  |  |  |  |  |  |
| **Assessment of Quality of Life 6D for adolescents (AQOL-6D)** | **Allen et al (2013)** | **Cronbach;s alpha: 0,50-0,86 (Allen et al. p.6)** | **Test retest reliability (ICCs=0.55–0.75),** | **no** | **no** | **Yes, confirmatory factor analyses.** | **yes, comparison to SF-36; Pearson -0,2 - -0,8, known groups, life satisfaction** | **no** |  | **no** |  |
| **Assessment of Quality of Life 6D for adolescents Mental Health Scale (AQOL-MHS)** | **Chavez et al (2013a)** |  |  |  |  |  |  |  |  | **reliability of change coefficient: 0,652-0,782, which is acceptable (Chavez et al. 2013a p.3195)** |  |
| **Assessment of Quality of Life 6D for adolescents Mental Health Scale (AQOL-MHS)** | **Chavez et al (2013b)** | **Cronbach's alpha:.80-.85 total scale 0,87 (Chavez et al. 2013b p.1331)** | **Test retest reliability (ICCs=.82-.90, total scale .89),** | **no** | **yes (Chavez et al. 2013b p.1333)** | **yes, exploratory factor analysis** | **convergent (family functioning and r=.59 and functional impairment (.60), discriminant (between community and clinical youth ), concurrent: comparison to YQOL-R & APGAR; dependent correlation 0,75** |  |  | **no** |  |

### Table 4. Chip

| **Version** | **Reference and instrument identification** | **Internal consistency** | **Reliability** | **Measurement error** | **Content validity** | **Structural validity** | **Hypotheses testing** | **Cross-cultural validity** | **Criterion validity** | **Responsive-ness** | **feasibility** |
| --- | --- | --- | --- | --- | --- | --- | --- | --- | --- | --- | --- |
| **CHIP-CE-PF** | **Riley_2004 The Parent Report Form of the CHIP-Child Edition: Reliability and Validity** | **yes, cronbachs alpha ranging from .79 to .88)** | **yes, test retest with ICC (between .36 (comfort) and .85)** |  |  | **yes, principle components analysis.** | **yes, differences in age gender, and poverty status, correlation with CHQ (.22 and .58) and CBCL (.66 correlation with risk avoidance)** |  |  |  | **yes, child rating about liking the questionnaire** |
| **CHIP-CE-PF** | **Riley_2007_A global measure of child health-related quality of life: reliability and validity of the Child Health and Illness Profile - Child Edition (CHIP-CE) global score** | **yes (of the five domain scores was 0.77, which is quite acceptable for group comparisons19. In contrast, if the α value is calculated using all 76 items in the five domains, α = 0.93,)** | **yes, test retest with ICC (.88)** |  |  | **yes, principle components analysis.** | **yes, known groups (between ADHD, Learning disorder, and healthy)** |  |  |  |  |
| **CHIP-CE-CRF** | **Estrada_2012_Reliability and validity of the Spanish version of the Child Health and Illness ProfileChild-Edition/Child Report Form (CHIP-CE/CRF)** | **yes, between .60 and .79 for the spanish version** | **yes, test retest with ICC between .69 and .80 for the spanish version.** |  |  | **yes, confirmatory factor analysis** | **yes, known groups (based on the CBCL, known differences (age and gender)** |  |  |  |  |
| **CHIP-CE_PF** | **Riley_2006_Validity of the health-related quality of life assessment in the ADORE study: Parent Report Form of the CHIP-Child Edition** | **yes, cronbachs alpha all above .70** |  |  |  | **yes, with** linear principal  factor model | **yes, comparison with other questionnaires (ADHDRS , SDQ==> moderate to high correlations)** |  |  |  |  |
| **CHIP-CE** | **Demirsoy_2016_Validity and reliability of a quality-of-life assessment instrument in children aged between 6 and 11 years** | **yes cronbachs alpha ( between .54 and .80)** |  |  | **yes** | **yes, confirmatory factor analysis** | **yes, in treatment vs not in treatment** |  |  |  |  |
| **CHIP-CE** | **Riley_2004_The Child Report Form of the CHIP-Child Edition: Reliability and Validity** | **yes cronbachs alpha between .70 and .82** | **yes, test retest with ICC (.63 to .76)** |  |  | **yes, principle components analysis.** | **yes, DIF analysis on age, gender, income** |  |  |  | **yes, time needed (21.4 minutes), extent and placement of missing items** |
| **CHIP-CE** | **Schacht_2011_Psychometric properties of the quality of life scale Child Health and Illness Profile-Child Edition in a combined analysis of five atomoxetine trials** | **yes, cronbachs alpha between .71 and .82** |  |  |  | **yes, factor analysis** | **yes, correltation with ADHD-RS (CHIP-CE total score: r = -0.345) except for the Risk avoidance domain (r = -0.517) and its sub-domains (individual risk avoidance r = -0.481, threats to achievement r = -0.463)** |  |  |  |  |
| **CHIP-AE** | **Alonso_2008_Validity of the Health Profile-Types of the Spanish Child Health and Illness Profile-Adolescent Edition (CHIP-AE)** |  |  |  |  |  | **yes, gender (girls more often in worst health profile),age (older lower health)** |  |  |  |  |
| **CHIP-AE** | **Rajmil_2003_The Spanish Version of the Child Health and Illness Profile-Adolescent Edition (CHIP-AE™)** | **yes, cronbachs alpha between .65 and .92** | **yes, test retest with ICC (between .57 and .93)** |  | **yes, expert team and focus groups** |  | **yes, age (older scored worse), gender (girls scored worse in discomfort and limitations of activities, lower self esteem and overall satisfaction with health) , or illness groups** |  |  |  |  |
| **CHIP-AE** | **STarfield_The Adolescent Child Health and Illness Profile: A Population-Based Measure of Health** | **yes between .42 and .93)** | **yes, test retest with ICC (between .48 and .93** |  |  | **yes, exploratory factor analysis** | **yes, known groups, known differences (age, gender(SES), and depression list (example, the correlation of the Self-Es- teem subdomain and a depression scale (CDI) was somewhat lower (- 0.40)) and agreement with parents (from 0.34 to 0.46 in the EB high school and from 0.45 to 0.54 in the EB middle school)** |  |  |  |  |
| **CHIP-AE** | **Estrada_2010_Reliability and validity of the Spanish version of the Child Health and Illness Profile (CHIP) Child- Edition, Parent Report Form (CHIP-CE/PRF)** | **Yes between .53 and .86** | **Yes, test retest between .46 and .85, and parent child agreement (ICC between 0.22-0.37)** |  |  |  | **Yes, Difference between gender, age eductation of parents, known groups based on CBCL** |  |  |  |  |
| **CHIP** | **Starfield_1993_adlolescent health status measurement development of the child health and illness profile** | **yes cronbachs alpha between .02 and .92** |  |  | **yes, expert panel** |  | **yes, known groups (accute,chronic, mentally ill, healthy** |  |  |  | **yes** |

Note. Starfield B, Bergner M, Ensminger M, et al. Adolescent Health-Status Measurement—Development of the Child Health and Illness Profile. Pediatrics 1993;91:430–5.

### Table 5. CHQ

| **Version** | **Reference and instrument identification** | **Internal consistency** | **Reliability** | **Measurement error** | **Content validity** | **Structural validity** | **Hypotheses testing** | **Cross-cultural validity** | **Criterion validity** | **Responsive-ness** | **feasibility** |
| --- | --- | --- | --- | --- | --- | --- | --- | --- | --- | --- | --- |
| **PF-50 &CF-87** | **Ng_2005_Preliminary Evidence on the Measurement Properties of the Chinese Version of the ChildHealth Questionnaire, Parent Form (CHQ-PF50) and Child Form (CHQ-CF87)** | **yes, cronbachs alpha ( parent version between .68 and .88, child version between .82 and .94)** |  |  | **yes, face validity** |  |  |  |  |  | **yes, ease of completion, and acceptability** |
| **PF-50 &CF-87** | **WaterS_1999-measuring health and wellbeing of children and adolescents: a preliminary compartice evaluation of the CHQ in australia** | **yes, cronbachs alpha ( (parent between .66 and .93, self between .63 and .90 )** |  |  | **yes, face validity** |  |  |  |  |  |  |
| **PF-50** | **Bae_2001_The Korean version of the Childhood Health Assessment Questionnaire (CHAQ) and the Child Health Questionnaire (CHQ)** | **yes, cronbachs alpha (between .66 and .9)** | **yes, test-retest with ICC ( between .6 and 1.0)** |  | **yes. parents tested understanding** |  | **yes, correlation with JIA core set variables (between -.2 and -.6) and difference between Juvinile artitis patients and healthy.** | **bekijkt naar koreanse situatie** |  |  |  |
| **PF-50** | **De inocencio_2001_The European Spanish version of the Childhood Health Assessment Questionnaire (CHAQ) and the Child Health Questionnaire (CHQ)** | **yes, cronbachs alpha (.7-.96)** | **yes, test-retest with ICC (between .2 and .9)** |  | **yes. parents tested understanding** |  | **yes, correlation with JIA core set variables (between 0.4 and -.2)and difference between Juvinile artitis patients and healthy.** |  |  |  |  |
| **PF-50** | **Haskes_2001_The Hebrew version of the Childhood Health Assessment Questionnaire (CHAQ) and the Child Health Questionnaire (CHQ)** | **yes, cronbachs alpha (between .4 and .91)** | **yes, test-retest with ICC (between -.1 (RP) and 1.0)** |  | **yes. parents tested understanding** |  | **yes, correlation with JIA core set variables (between -.5 and -.2 ) and difference between Juvinile artitis patients and healthy.** |  |  |  |  |
| **PF-50** | **Hepner_2002_Confirmatory Factor Analysis of the Child Health Questionnaire-Parent Form 50 in aPredominantly Minority Sample** |  |  |  |  | **yes, confirmatory factor analysis** |  |  |  |  |  |
| **PF-50** | **Hofer_2001_he Swiss German and Swiss French versions of the Childhood Health Assessment Questionnaire (CHAQ) and the Child Health Questionnaire (CHQ)** | **yes, cronbachs alpha (between .69 and.92)** | **yes, test-retest with ICC (between .4 and 1.0)** |  | **yes. parents tested understanding** |  | **yes, correlation with JIA core set variables (between -.3 and -.1) and difference between Juvinile artitis patients and healthy.** |  |  |  |  |
| **PF-50** | **Joos_2001_The Belgian-Flemish version of the Childhood Health Assessment Questionnaire (CHAQ) and the Child Health Questionnaire (CHQ)** | **yes, cronbachs alpha (overal.93, range .28-.99)** | **yes, test-retest with ICC (range -.1 to .9 with poor reproducibiltu of REB, PE, PT)** |  | **yes. parents tested understanding** |  | **yes, correlation with JIA core set variables (between -.7 and -.1) and difference between Juvinile artitis patients and healthy.** |  |  |  |  |
| **PF-50** | **Landgraf_1998_Canadian-French, German and UK Versions of the Child Health Questionnaire:Methodology and Preliminary Item Scaling Results** | **yes, cronbachs alpha between .60 and .88)** |  |  |  |  |  |  |  |  |  |
| **PF-50** | **Machado_2001_The Brazilian version of the Childhood Health Assessment Questionnaire (CHAQ) and the Child Health Questionnaire (CHQ)** | **yes, cronbachs alpha (between .4 and .94)** | **yes, test-retest with ICC (between .2 and. 9)** |  | **yes. parents tested understanding** |  | **yes, correlation with JIA core set variables (between-.0 and-.6) and difference between Juvinile artitis patients and healthy.** |  |  |  |  |
| **PF-50** | **Mihaylova_2001_The Bulgarian version of the Childhood Health Assessment Questionnaire (CHAQ) and the Child Health Questionnaire (CHQ)** | **yes, cronbachs alpha (between .64 and .95)** |  |  | **yes. parents tested understanding** |  | **yes, correlation with JIA core set variables (between-.1 and -.7)) and difference between Juvinile artitis patients and healthy.** |  |  |  |  |
| **PF-50** | **Miranda_2001_The Chilean version of the Childhood Health Assessment Questionnaire (CHAQ) and the Child Health Questionnaire (CHQ)** | **yes, cronbachs alpha (between .8 and .95)** | **yes, test-retest with ICC (between .4 and 1.0)** |  | **yes. parents tested understanding** |  | **yes, correlation with JIA core set variables (between-.1eand -.7)) and difference between Juvinile artitis patients and healthy.** |  |  |  |  |
| **PF-50** | **Nugent_2001_The British version of the Childhood Health Assessment Questionnaire (CHAQ) and the Child Health Questionnaire (CHQ)** | **yes, cronbachs alpha (between .8 and .96)** | **yes, test-retest with ICC (between .6 and 1.0)** |  | **yes. parents tested understanding** |  | **yes, correlation with JIA core set variables (between-.2 and -.8)) and difference between Juvinile artitis patients and healthy.** |  |  |  |  |
| **PF-50** | **Orban_2001_The Hungarian version of the Childhood Health Assessment Questionnaire (CHAQ) and the Child Health Questionnaire (CHQ)** | **yes, cronbachs alpha (between .7 and .93)** | **yes, test-retest with ICC (between .2 (PT) and .9)** |  | **yes. parents tested understanding** |  | **yes, correlation with JIA core set variables (between0 and -.6)) and difference between Juvinile artitis patients and healthy.** |  |  |  |  |
| **PF-50** | **Ozdogan_2001_The Turkish version of the Childhood Health Assessment Questionnaire (CHAQ) and the Child Health Questionnaire (CHQ)** | **yes, cronbachs alpha (between .66 and .96)** | **yes, test-retest with ICC ( between -.4 and .6)** |  | **yes. parents tested understanding** |  | **yes, correlation with JIA core set variables (between-.2 and -.7)) and difference between Juvinile artitis patients and healthy.** |  |  |  |  |
| **PF-50** | **Pagava_2001_The Georgian version of the Childhood Health Assessment Questionnaire (CHAQ) and the Child Health Questionnaire (CHQ)** | **yes, cronbachs alpha (between .73 and .96)** | **yes, test-retest with ICC (.8 and 1.0)** |  | **yes. parents tested understanding** |  | **yes, correlation with JIA core set variables (between-.2 and -.6)) and difference between Juvinile artitis patients and healthy.** |  |  |  |  |
| **PF-50** | **Pelkonen_2001_The Finnish version of the Childhood Health Assessment Questionnaire (CHAQ) and the Child Health Questionnaire (CHQ)** | **yes, cronbachs alpha (between .52 and .91)** | **yes, test-retest with ICC (between .4 and 1.0)** |  | **yes. parents tested understanding** |  | **yes, correlation with JIA core set variables (between -.1 and -.7))) and difference between Juvinile artitis patients and healthy.** |  |  |  |  |
| **PF-50** | **Pouchot_2001_The French version of the Childhood Health Assessment Questionnaire (CHAQ) and the Child Health Questionnaire (CHQ)** | **yes, cronbachs alpha (.67 to .94)** | **yes, test-retest with ICC (between .64 and .85)** |  | **yes. parents tested understanding** |  | **yes, correlation with JIA core set variables (between -.21 and -.50) and difference between Juvinile artitis patients and healthy.** |  |  |  |  |
| **PF-50** | **Pratsidou-Gertsi_2001_The Greek version of the Childhood Health Assessment Questionnaire (CHAQ) and the Child Health Questionnaire (CHQ)** | **yes, cronbachs alpha (between .6 and .95)** | **yes, test-retest with ICC (between -.3 (RP and .9)** |  | **yes. parents tested understanding** |  | **yes, correlation with JIA core set variables (between-.3 and -.5) and difference between Juvinile artitis patients and healthy.** |  |  |  |  |
| **PF-50** | **Raat_2002_Reliability and validity of comprehensive health status measures in children: The Child Health Questionnaire in relation to the Health Utilities Index** | **yes, cronbachs alpha ranging from .36 to .96)** | **yes, test-retest with ICC (between -.08 (role functionphusical) and .84)** |  |  |  | **yes, known groups (asthma, ADHD, dutch schools) (number of chronic conditions per child, number of cisits to physician last year)and comparison with HUI2 ( correlations between .26 and.49)** |  |  |  | **yes, missing items (max2%)** |
| **PF-50** | **Rentz_2005_Psychometric validation of the child health questionnaire (CHQ) in a sample of children and adolescents with attention-deficit/hyperactivity disorder** | **yes, cronbachs alpha (ranging from .53 to .91)** |  | **yes, using SEM (raning from 3.90 to 12.62)** |  |  | **yes known groups (conners en CGI en ADHD-RS) construct and discriminant validity** |  |  | **yes, comparison with improvement in clinical status after 10 weeks** |  |
| **PF-50** | **Ruperto_2001_The Italian version of the Childhood Health Assessment Questionnaire (CHAQ) and the Child Health Questionnaire (CHQ)** | **yes, cronbachs alpha (between .6 (GH) and .95)** | **yes, test-retest with ICC (between .4 and .8)** |  | **yes. parents tested understanding** |  | **yes, correlation with JIA core set variables (between-.3 and -.6) and difference between Juvinile artitis patients and healthy.** |  |  |  |  |
| **PF-50** | **Susic_2001_The Serbian version of the Childhood Health Assessment Questionnaire (CHAQ) and the Child Health Questionnaire (CHQ)** | **yes, cronbachs alpha (between .64 and .95)** | **yes, test-retest with ICC (between .3 (GGH) and 1.0)** |  | **yes. parents tested understanding** |  | **yes, correlation with JIA core set variables (between-.1 and -.7) and difference between Juvinile artitis patients and healthy.** |  |  |  |  |
| **PF-50** | **Waters_2000_The Child Health Questionnaire in Australia: reliability, validity and population means** | **yes, cronbachs alpha (between .19 general health and .87)** |  |  | **yes** |  | **yes, differences between gender, and age** |  |  |  |  |
| **PF-50** | **Waters_2000_the parent form child health questionnaire in australia: comparison of reliability, validity, structure and norms** | **yes, cronbachs alpha (between .60 and .94)** | **yes, test-retest with ICC (two week .49 and .78)** |  |  | **yes, exploratory factor analysis** | **yes, idifferences between males and females,** |  |  |  | **geeft zelf aan dat het concurrent validity is, maar is niet want known groups** |
| **PF-50** | **Wulffraat_2001_The Dutch version of the Childhood Health Assessment Questionnaire (CHAQ) and the Child Health Questionnaire (CHQ)** | **yes, cronbachs alpha (betweem. 76 and .97)** | **yes, test-retest with ICC ( between .5 and. 9)** |  | **yes. parents tested understanding** |  | **yes, correlation with JIA core set variables (between -.1 and -.8) and difference between Juvinile artitis patients and healthy.** |  |  |  |  |
| **PF-28** | **Raat_2005_Reliability and validity of the short form of the child health questionnaire for parents (CHQ-PF28) in large random school based and general population samples** | **yes, cronbachs alpha (all above .70)** | **yes, test-retest with ICC (from .14 to .78)** |  |  |  | **yes, known groups (no conditions, asthma, headaches, problems hearing), between VAS scale ( between .15 and .35)** |  |  |  | **yes, response rates (70 % of children, 71% of parents) between 0 and 1.5% missing items)** |
| **CF-87 (online version)** | **Raat_2007_Feasibility, reliability, and validity of adolescent health status measurement by the Child Health Questionnaire Child Form (CHQ-CF): internet administration compared with the standard paper version** | **yes, cronbachs alpha (between .69 and .92)** |  |  |  |  | **yes known groups (number of reported conditions)** |  |  |  | **yes, participation rate 87%, missing items between 0 and 1.89%)** |
| **CF-87** | **Helseth_2006_Health-related quality of life in a Norwegian sample of healthy adolescents: Some psychometric properties of CHQ-CF87-N in relation to KINDL-N** | **yes, cronbachs alpha (between .65 and .88)** |  |  |  | **yes, exploratory factor analysis** | **yes, comparison with the KINDL (total kindl to scales of CHQ between .16 (RB) and .72 (MH) correlations** |  |  |  |  |
| **CF-87** | **Hosli_2007_self report form of the child health questionnaire in a dutch adolescent pupilation** | **yes, cronbachs alpha (between .79 and .91)** |  |  |  | **yes, confirmatory factor analysis** | **yes, known groups (chronic illness, noncronic, any common, important life event) and correlation with the TACQOL and HUI3 scale (correlations between .10 to .61)** |  | **yes, concurrent validity with the TACQOL and HUI** |  |  |
| **CF-87** | **Landgraf_1997_Functional status and well-being of children representing three cultural groups: Initial selfreports using the chq-cf87** | **yes, cronbachs alpha (between .63 (GH) and .89)** |  |  |  |  | **yes, known group validity (between school sample, ADHD, and end stage renal disorder), age, and gender)** |  |  |  |  |
| **CF-87** | **Raat_2002_Reliability and validity of the child health questionnaire-child form (CHQ-CF87) in a Dutch adolescent population** | **yes, cronbachs alpha (between .56 and .90)** | **yes, test-retest with ICC between .25 and .40)** |  |  |  | **yes known groups (between number of chronic conditions)** |  |  |  | **yes, missing items (between 0 and 1.6%)** |
| **CF-80** | **Waters_2001_The Health and Well-being of Adolescents: A School-based Population Study of the Self-report Child Health Questionnaire** | **yes, cronbachs alpha (between .72 and .90 for the 87 items, for the 80 item between .79 and .92)** |  |  |  |  | **yes,differences between males and females (females scored ower for mental health self esteem general health family cohesion and change in health) and age (QOL decreased with age) and known health conditions** |  |  |  |  |

*Note. The CHQ was developed by Landgraf, J. M., Abetz, L., & Ware, J. E. (1996). The CHQ User’s Manual. 1st ed., Boston: The Health Institute, New England Medical Center.*

### Tabel 6. Comprehensive health status classification system -preschool

| **Reference and instrument identification** | **Internal consistency** | **Reliability** | **Measurement error** | **Content validity** | **Structural validity** | **Hypotheses testing** | **Cross-cultural validity** | **Criterion validity** | **Responsive-ness** | **feasibility** |
| --- | --- | --- | --- | --- | --- | --- | --- | --- | --- | --- |
| Pilot tested: Saigal et al. (2005) Psychometric evidence: Saigal et al. (2005) | no | yes, intrarater reliability of parental assessment kappa between ..38 (thinking and problem solving) and 1.00, interrater reliability between parent and clinician kappas between .47 and.66 | no | no | no | yes, between VLBW and term cohort, and between the BSID-II and the VABS | no | no | no |  |

### Tabel 7. CHUd9

| **Version** | **Reference and instrument identification** | **Internal consistency** | **Reliability** | **Measurement error** | **Content validity** | **Structural validity** | **Hypotheses testing** | **Cross-cultural validity** | **Criterion validity** | **Responsive-ness** | **feasibility** |
| --- | --- | --- | --- | --- | --- | --- | --- | --- | --- | --- | --- |
|  |  |  |  |  |  |  |  |  |  |  |  |
|  | **Canaway_2013_Measuring preference-based quality of life in children aged 6–7 years: a comparison of the performance of the CHU-9D and EQ-5D-Y—the WAVES Pilot Study** |  |  |  |  |  | **yes, known groups (healthy compared to less healthy) and comparability with EQ-5D (higher utility scores on the CHU9D (. 86compated to .72) (agreement ranged from 70.8 to 89.6) ,pedsql** |  |  |  | **yes, complete questionnaires (0% missing), and acceptibility (7% of children rated poor understanding** |
|  | **Dam-petersen_2017_Measuring Health-Related Quality of Life in Adolescent Populations: An Empirical Comparison of the CHU9D and the PedsQLTM 4.0 Short Form 15** |  |  |  |  |  | **yes, agreement with pedsql (based on ICC=.77 r = .63 ), scores were lowerer for those with known health differences. Higher SES was associated with higher QOL** |  |  |  |  |
|  | **Frew_2015_Is utility-based quality of life associated with overweight in children? Evidence from the UK WAVES randomised controlled study** |  |  |  |  |  | **yes, agreement with pedsql (above and below mean score) and correlation Rs = .47, en different weight groups (no differences), different etnic groups** |  |  |  |  |
|  | **Furber_2015_The validity of the Child Health Utility instrument (CHU9D) as a routine outcome measure for use in child and adolescent mental health services** | **yes, cronbachs alpha ( .78)** |  |  |  | **yes face validity** | **yes, convergence with SDQ (moderate correlation r =-.49)** |  |  |  | **yes, practicality assessed by qualitative infrmation collected by interviewers)** |
|  | **Ratcliffe_2012_An assessment of the construct validity of the CHU9D in the Australian adolescent general population** |  |  |  |  |  | **yes convergence with HUI (ICC indicated moderate agreement ), difference with age, gender and SES** |  |  |  | **yes , completion rates (70%), time taken to complete (between 1 and 85 minutes, respondents rating of difficultu and comments** |
|  | **Stevens_2012_Measuring andValuingHealthBenefitsforEconomicEvaluationin Adolescence: AnAssessmentofthePracticality andValidityoftheChild Health Utility9DintheAustralianAdolescentPopulation** |  |  |  | **yes, face validity** |  | **yes, discriminate between those with health issues and without, convergene with kidscreen-10 (divided in groups), age (decreasing with age) and gender (no differences)** |  |  |  | **yes, practicality (time for completion, and competion rates** |
|  | **Xu_2014_Measuring and Valuing Health-Related Quality of Life among Children and Adolescents in Mainland China – A Pilot Study** |  |  |  |  |  | **yes, comparison with self raported health status** |  |  |  |  |

Note. StevensKJ.Workingwithchildrentodevelopdimensionsfora

preference-based,generic,pediatrichealth-relatedquality-of-life

measure.QualHealthRes2010;20:340–51.

### Tabel 8. Child quality of life questionnaire (CQOL)

| **Reference and instrument identification** | **Internal consistency** | **Reliability** | **Measurement error** | **Content validity** | **Structural validity** | **Hypotheses testing** | **Cross-cultural validity** | **Criterion validity** | **Responsive-ness** | **feasibility** |
| --- | --- | --- | --- | --- | --- | --- | --- | --- | --- | --- |
| Pilot tested: Graham et al. (1997) Psychometric evidence: Graham et al. (1997) | Cronbach's alpha: 0,81-0,86 for mothers and between .85 and .87 for children(p. 660) | test-retest: 0,4-0,7 for both children and mothers (correlation of combined function scores, p. 660), intra-rater correlation: 0,57 (p. 663) | no | face validity (p. 663) | no | Yes, correlation with the CGAS (r=.64), and known groups between chronic disorders, psychiatric disorders, and controls, | no |  | no |  |

### Table 9. EQ-5D

| **Version** | **Reference and instrument identification** | **Internal consistency** | **Reliability** | **Measurement error** | **Content validity** | **Structural validity** | **Hypotheses testing** | **Cross-cultural validity** | **Criterion validity** | **Responsive-ness** | **feasibility** |
| --- | --- | --- | --- | --- | --- | --- | --- | --- | --- | --- | --- |
| **EQ-5d-Y** | **canaway_2013_Measuring preference-based quality of life in children aged 6–7 years: a comparison ofthe performance of the CHU-9D and EQ-5D-Y—the WAVES Pilot Study** |  | **yes, test retest with kappa between .17 and .48)** |  |  |  | **yes, known groups and comparability with pedsql and CHUD9 higher utility score in the CHU9D)** |  |  |  | **yes, 0% missing items, and 7.1% children rated the instrument as poor** |
| **EQ-5d-Y** | **bouwmans_2014_Validity and responsiveness of the EQ-5D and the KIDSCREEN-10 in children with ADHD** |  |  |  |  | **yes, PCA** | **yes, construct validity; correlation with kidscreen (correlations between total score was strong, but not on separate items),** |  |  | **yes, comparison between those responding to treatment and thos not responding** |  |
| **EQ-5d-Y** | **Burstrom_2010_Testing a Swedish child-friendly pilot version of the EQ-5D instrument—initial results** |  |  |  |  |  | **yes, known groups ( based on SES, previous illness)** |  |  |  | **yes, no missing items comprehension and acceptibility (as observed by interviewing nurse)** |
| **EQ-5D-3L** | **Byforde_2013_The validity and responsiveness of the EQ-5D measure of health-related quality of life in an adolescent population with persistent major depression** |  |  |  |  |  | **yes, agreement with clinical measures (inpatient admissions (nodifferences), suicide attempts (nodifferences), number of comorbid mental disorders, and severity of illness measured by the CGAS (nodifferences), MFQ, CDRS and HONOSCA, differences between age and gender (nodifferences with age and gender)** |  | **yes, agreement with HONOSCA** | **yes, agreement with CGI over time** | **yes, acceptibility** |
| **EQ-5d-Y** | **Chen_2015_Assessing theHealth-RelatedQualityofLifeofAustralian Adolescents: AnEmpiricalComparisonoftheChildHealth Utility 9DandEQ-5D-Yinstruments** |  |  |  |  |  | **yes comparison with FAS scale (instrument is able to differentiate between age, sex, and GAS subgroups), and CHUd9 (icc =.80)** |  |  |  |  |
| **EQ-5d-Y** | **Kind_2015_Can adult weights be used to value child health states? Testing the influence of perspective in valuing EQ-5D-Y** |  |  |  | **yes, face validity** |  |  |  |  |  |  |
| **EQ-5d-Y** | **Lereeya_2016_The student resilience survey: psychometric validation and associations with mental health** | **yes (cronbachs alpha is .65)** |  |  |  |  |  |  |  |  |  |
|  | **Matza_2005_Parent-Proxy EQ-5D Ratings of Children with Attention-Deficit Hyperactivity Disorder in the US and the UK** |  |  |  |  |  | **yes, known groups (with and without problems), correlation with ADHD RS ( between -.31 and -.27 (index scores)) and CHQ-PF50 scale (index scores between -.11 and .64and** |  |  |  |  |
| **EQ-5d-Y** | **Ravens-sieberer_2010_Feasibility, reliability, and validity of the EQ-5D-Y: results from a multinational study** |  | **yes, test retest with kappa (between 69.8 and 99.7%) and ICC (.82-.83)** |  |  |  | **yes, known groups (chronic conditions good health and mental health problems based on SDQ), KS-127 ( between -.41 and -.52) life satisfactcion ladder (correlation .33-.56)** |  |  |  | **yes , complete data in 91-100% of samples** |
| **EQ-5d-Y** | **Robles_2015_Development of the web-based Spanish and Catalan versions of the Euroqol 5D-Y (EQ-5D-Y) and comparison of results with the paper version** |  | **yes, test retest with kappa (between 89 and 97%) and ICC (.82-.86)** |  |  |  | **yes known groups (self reported health based on SDQ )** |  |  |  | **yes ceiling effects (66.3%) floor effects (.1%), participantion rate 77%** |
| **EQ-5d-Y** | **Scot_2017_The use of the EQ-5D-Y health related quality of life outcome measure in children in the Western Cape, South Africa: psychometric properties, feasibility and usefulness - a longitudinal, analytical study** |  | **yes, test retest with kappa and ICC (.765)** |  |  |  | **yes known group base don SDQ, scores on PEDSQL, WeeFIM and FPS** |  |  | **yes, responsiveness over time** | **yes** |
| **EQ-5d-Y** | **secnic_2005_Health State Utilities for Childhood Attention- Deficit/Hyperactivity Disorder Based on Parent Preferences in the United Kingdom** |  |  |  |  |  |  |  |  |  |  |
| **EQ-5d** | **Stochl_2013_Usefulness of EQ-5D for evaluation of health-related quality of life in young adults withfirst-episode psychosis** |  |  |  |  |  |  |  |  |  |  |
| **EQ-5d-Y** | **Wille_2010_Development of the EQ-5D-Y: a child-friendly version of the EQ-5D** |  |  |  | **yes, cognitive interviews** |  |  |  |  |  |  |
| **EQ-5d-Y** | **Willems_2009_Using EQ-5D in children with asthma, rheumatic disorders, diabetes, and speech/language and/or hearing disorders** |  | **yes test retest with ICC between -.25 and 1.00 (lowest ICC in children with Asthma** |  |  |  | **yes, discriminant validity (between groeps with asthma diabetes rheumatic disorder, speech or hearing disorder) and correlation with the TACQOL ( low to moderate correlations)** |  |  |  | **Yes, practicallity** |

Note. Developed by Development of the EQ-5D-Y: a child-friendly version of the EQ-5D

Wille N, Badia X, Bonsel G, Burstrom K, Cavrini G, Devlin N, Egmar AC, Greiner W, Gusi N, Herdman M, Jelsma J

Qual Life Res 2010 Aug;19(6):875-892

### Tabel 10. Generic children’s quality of life questionnaire (GCQ)

| **Reference and instrument identification** | **Internal consistency** | **Reliability** | **Measurement error** | **Content validity** | **Structural validity** | **Hypotheses testing** | **Cross-cultural validity** | **Criterion validity** | **Responsive-ness** | **feasibility** |
| --- | --- | --- | --- | --- | --- | --- | --- | --- | --- | --- |
| **Psychometric evidence: Collier et al. (2000)** | **Cronbach's alpha: 0,75 total scale, .74 for self perceived scores and .78 for QoL** | **Cron** | **no** | **Yes, face validity** | **no** | **Yes difference between age (r=-.02) and geographical location and sex (no differences)** | **no** | **no** | **no** |  |

Note.

### Table 11. HUI

| **Version** | **Reference and instrument identification** | **Internal consistency** | **Reliability** | **Measurement error** | **Content validity** | **Structural validity** | **Hypotheses testing** | **Cross-cultural validity** | **Criterion validity** | **Responsive-ness** | **feasibility** |
| --- | --- | --- | --- | --- | --- | --- | --- | --- | --- | --- | --- |
| Hui3 | Oluboyede_2013_Measuring health outcomes of adolescents: report from a pilot study |  |  |  |  |  |  |  |  |  | yes, completion rates (73.47), and understanding (less than the EQ-5D) |

Note. Developed by Feeny, David, William Furlong, Michael Boyle, and George W. Torrance, "Multi-Attribute Health Status Classification Systems:  Health Utilities Index." PharmacoEconomics, Vol 7, No 6, June, 1995, pp 490-502.

### Tabel 12. ITQOL

| **Reference and instrument identification** | **Internal consistency** | **Reliability** | **Measurement error** | **Content validity** | **Structural validity** | **Hypotheses testing** | **Cross-cultural validity** | **Criterion validity** | **Responsive-ness** | **feasibility** |
| --- | --- | --- | --- | --- | --- | --- | --- | --- | --- | --- |
| **Raat et al. (2007),** | **Cronbach's alpha between .72 and .94** | **test-retest ICC between .01 (change in health general population) and . 82)** | **no** | **no** | **no** | **yes, correaltion with TAPQOL and comparison between subgroups with many medical conditions(Raat et al. p.451)** | **no** | **no** | **no** | **no** |
| **Spuijerbroek et al. 2011** | **no** | **no** | **no** | **no** | **no** | **Yes, difference between 5 clinical subgroups, influence of gender and age** | **no** |  | **yes** | **no** |
| **Landgraf et al (2012)** | **Cronbach's alpha: 0,.61 and .94** | **no** | **no** | **no** | **no** | **between groups with abdomial pain, and without and burn victoms discriminant validity 91-100%** | **no** | **no** | **no** | **no** |

Note.

### Table13. Kidscreen

| **Version** | **Reference and instrument identification** | **Internal consistency** | **Reliability** | **Measurement error** | **Content validity** | **Structural validity** | **Hypotheses testing** | **Cross-cultural validity** | **Criterion validity** | **Responsive-ness** | **feasibility** |
| --- | --- | --- | --- | --- | --- | --- | --- | --- | --- | --- | --- |
| **KIDSCREEN-10** | **Bouwmans_2014_Validity and responsiveness of the EQ-5D and the KIDSCREEN-10 in children with ADHD** |  |  |  |  | **yes, principle component analysis with varimax rotation** | **yes, EQ5D and kidscreen-10 (correlation total score .556)** |  |  | **Yes, children responsive to treatment compared to not responsive** |  |
| **KIDSCREEN-10** | **Chen_2014_From KIDSCREEN-10 to CHU9D: creating a unique mapping algorithm for application in economic evaluation** |  |  |  |  |  | **yes, correlation with CHU9D (between .175 and .447)** |  |  |  |  |
| **KIDSCREEN-10** | **deMatos_2012_Health-Related Quality of Life in Portuguese Children and Adolescents** | **yes, cronbachs alpha (.78)** |  |  |  | **yes, confirmatory factor analysis** | **yes, construct validity (comparison over nationality, age, SES ( no differences)** |  |  |  |  |
| **KIDSCREEN-10** | **Nik-azin_2014_The Health-Related Quality of Life Index KIDSCREEN-10: Confirmatory Factor Analysis, Convergent Validity and Reliability in a Sample of Iranian Students** | **yes, cronbachs alpha (.80)** | **yest, test retest reliability with ICC(.86)** |  |  | **yes, confirmatory factor analysis** | **yes, comparison with PEDSQL (moderate correlation), GHQ (relatively strong (except somatic symptoms) and negative correlation), MSLSS (correlation coefficients between KIDSCREEN-10 and MSLSS dimensions were not significant, except for Perceived qol dimension of MSLSS in which the correlation coefficient obtained was significant and correlation was strong)** |  | **Yes correltation with the KS-52 (Moods and Emotions dimension of KIDSCREEN-52 had the greatest correlation with KIDSCREEN-10, while the correlations for the dimensions of Physical Well-being, Psychological Well-being, Moods and Emotions, Self-Perception, Autonomy, Parents Relations and Home Life, and School Environment, was relatively strong, and the correlations for Social Support and Peers, Social Acceptance (Bullying), and Financial Resources dimensions were moderate.)** |  |  |
| **KIDSCREEN-10** | **Ravens-sieberer_2010_Reliability, construct and criterion validity of the KIDSCREEN-10 score: a short measure for children and adolescents’ well-being and health-related quality of life** | **yes, cronbachs alpha ((8–11-year olds: 0.79; 12–18-year olds: 0.81))** | **yest, test retest reliability with ICC (8–11-year olds: 0.64; 12–18-year olds: 0.69)** |  |  |  | **yes, correlation between KIDSCREEN-10 self and parent proxy reports was r = 0.54, cPedsQL scales and summary measure (0.57), the CHIP satisfaction scale (0.63), and the YQOL-S perceptual scale (0.61), known groups validity (based on SDQ)** |  | **yes, comparison with kidscreen 52 (self-report and parent report versions and scales of the KIDSCREEN-52 ranged from 0.24 to 0.72 and 0.27 to 0.72)** |  |  |
| **KIDSCREEN-10 & KIDSCREEN-52** | **Rajmil_2014_Comparison of the Web-Based and Digital Questionnaires of the Spanish and Catalan Versions of the KIDSCREEN-52** | **yes, cronbachs alpha (paper version .70 to .87, web based .70-.88)** | **yes, interrater reliability (comparison between web and paper based ICC 0.75 to 0.87)** |  |  |  | **yes, comparison between web and paper based, known group (based on SDQ scores)** |  |  |  |  |
| **Kidscreen-10, KIDSCREEN-27** | **Nezu_2016_Reliability and validity of Japanese versions of KIDSCREEN-27 and KIDSCREEN-10 questionnaires** | **yes, cronbachs alpha (KS-27 between .78 and.87, ks 10 .84)** | **yes, intra-raterreliability and test-retest reliability with ICCKs-27 between 0.73–0.79, KS-10 .79) (parent child)** |  |  |  | **yes, pearson correltations with PedsQL (ks.27 between .33 and .45, ks-10 .46)** |  | **yes, correspondence ks27 with ks 52 (0.71 to 0.98), correspondence between ks-27 and ks10 between .61 and .82)** |  |  |
| **KIDSCREEN-10, KIDSCREEN-52** | **Haraldstad_2011_Health related quality of life in children and adolescents: Reliability and validity of the Norwegian version of KIDSCREEN-52 questionnaire, a cross sectional study** | **yes, cronbachs alpha (between .81 and .89)** |  |  |  | **yes, confirmatory factor analysis** | **yes, comparison with KINDL (physical well-being, psychological well-being, self-perception, parent relation and home life, social support and peers, and school environment correlated considerably with similar KINDL scales, such as physical well-being, emotional wellbeing, self-esteem, family, friends, and school)** |  |  |  |  |
| **KIDSCREEN-27** | **Andersen_2016_Psychometric properties of the Norwegian version of the Kidscreen-27 questionnaire** | **yes, cronbachs alpha (between .77 and .82)** | **yest, test retest reliability with ICC (between .71 (autonomy and parents) and .81 (school environment))** |  |  | **yes, confirmatory factor analysis** | **yes, agreement with Cantrils life satisfaction ladder (significant Rs between .31 and .59)** |  |  |  |  |
| **KIDSCREEN-27** | **Bagheri_2014_Assessing Whether Measurement Invariance of the KIDSCREEN-27 across Child-Parent Dyad Depends on the Child Gender: A Multiple Group Confirmatory Factor Analysis** |  |  |  |  | **yes, Multiple group categorical confirmatory factor analysis (MGCCFA)** |  |  |  |  |  |
| **KIDSCREEN-27** | **Berman_2016_Children’s Quality of Life Based on the KIDSCREEN-27: Child Self-Report, Parent Ratings and Child-Parent Agreement in a Swedish Random Population Sample** |  |  |  |  |  | **yes, parent child agreement (14 items (51.9%) had slight agreement, 12 (44.4%) had fair agreement, and one (3.7%) had agreement less than chance. Items in the Social support and peer relations dimension showed relatively low concordance, with PABAK-OS ranging from 0.13 to 0.31.)** |  |  |  |  |
| **KIDSCREEN-27** | **Erhart_2006_Health-related quality of life instruments and individual diagnosis - a new area of application** | **yes, cronbachs alpha (ranged from .78 to .84)** |  |  |  | **yes, principal componant analysis** | **yes, ability to derect mental health problem with AUC** |  |  |  |  |
| **KIDSCREEN-27** | **Jafari_2012_Item and response-category functioning of the Persian version of the KIDSCREEN-27: Rasch partial credit model** | **yes, cronbachs alpha (child self between .73 and .85, parent between .75 and .83))** |  |  |  |  |  |  |  |  |  |
| **KIDSCREEN-27** | **Meade_2015_Health-related quality of life in a sample of Australian adolescents: gender and age comparison** |  |  |  |  |  | **yes, gender (boys score higher than girls), age (younger subjects score higher)** |  |  |  |  |
| **KIDSCREEN-27** | **Ravens-Sieberer_2007_The KIDSCREEN-27 quality of life measure for children and adolescents: psychometric results from a cross-cultural survey in 13 European countries** |  | **yest, test retest reliability with ICC (between.61 and .74)** |  |  |  | **yes, comparison withCHIP (.39-.62), YQOL (.37-.63), HBSC (-.25--.52) and known groups (based on SDQ), age (younger scores higher) and gender** |  | **yes, comparison with kidscreen 52 (Correlations between KIDSCREEN-27 scales and scales of the KIDSCREEN-52 measuring similar dimensions ranged from 0.71 to 0.96)** |  |  |
| **KIDSCREEN-27** | **Robitail_2007_Testing the structural and cross-cultural validity of the KIDSCREEN-27 quality of life questionnaire** | **yes, cronbachs alpha (.78 and .84)** |  |  |  | **yes, exploratory factor analysis, confirmatory factor analysis** |  | **yes, Differential item functioning (DIF) analyses based on IRT modeling** |  |  |  |
| **KIDSCREEN-27** | **Stevanovic_2015_A cross-cultural study to assess measurement invariance of the KIDSCREEN-27 questionnaire across Serbian and Iranian children and adolescents** |  |  |  |  |  |  | **niet nl, maar serbian en iranian** |  |  |  |
| **KIDSCREEN-27 (online version)** | **Lloyd_2011_Kids’ Life and Times: using an Internet survey to measure children’s health-related quality of life** | **yes, cronbachs alpha (between .76 and .84)** |  |  |  | **yes, exploratory factor analysis** | **yes, gender (boys have higher scores than girls)** |  |  |  | **yes, item non response** |
| **KIDSCREEN-52** | **Berra_2013_Reliability and validity of the KIDSCREEN-52 questionnaire to measure health related quality of life in the 8 to 18 year-old Argentinean population** | **yes, cronbachs alpha (between .65 and .88)** |  |  |  | **yes, confirmatory factor analysis** | **yes, construct validity (comparison with FAS scale) and differences with age and gender (Lower scores with age, and boys higher scores)** |  |  |  | **yes, acceptibility based on missing values (between 2-4,5%) floor (between .05-2.1%) and ceiling effects( between 0-31.4%)** |
| **KIDSCREEN-52** | **Berra_2007_Methods and representativeness of a European survey in children and adolescents: the KIDSCREEN study** |  |  |  |  |  |  |  |  |  | **yes, cooperation (between 42% and 91.2%)** |
| **KIDSCREEN-52** | **Clark_2015_Youth With Autism Spectrum Disorders: Self- and Proxy-Reported Quality of Life and Adaptive Functioning** | **yes, cronbachs alpha (between .72 and .89 for child-report, between .78 and .92 for parent-report)** | **yes, intra-raterreliability with ICC (parent child) (ICC between -.17 and .66)** |  |  |  | **yes, comparison with ABAS-II (correlations were low)** |  |  |  |  |
| **KIDSCREEN-52** | **Gaspar_2010_Parent–child perceptions of quality of life: Implications for health intervention** | **yes, cronbachs alpha (ranged from .60 to .88)** |  |  |  |  | **yes, age (higher age, lower score), gender (boys score higher)** |  |  |  |  |
| **KIDSCREEN-52** | **Nezu_2015_Reliability and validity of the Japanese version of the KIDSCREEN-52 health-related quality of life questionnaire for children/adolescents and parents/proxies** | **yes, cronbachs alpha (self report between .68 and .93, parent between .71 and .94))** | **yes, intra-raterreliability(parent child, ICC between ) and test-retest reliability with ICC (self between .81 and .87, parent between (.56 and .75)** |  |  | **yes, principal componants** | **yes, comparison with PEDSQL (correlation self between .36 and .52, correlation parent between .22 and .56)** |  |  |  |  |
| **KIDSCREEN-52** | **Parizi_2014_Psychometric properties of KIDSCREEN health-related quality of life questionnaire in Iranian adolescents** | **yes, cronbachs alpha (between .60 and .93)** |  |  |  | **yes, confirmatory factor analysis** |  |  |  |  |  |
| **KIDSCREEN-52** | **Guedes_2011_Translation, cross-cultural adaptation and psychometric properties of the kidscreen-52 for the brazilian population** | **yes, cronbachs alpha (self between .73 and .89, parent between .75 and .86)** |  |  | **yes, expert panel** | **yes, exploratory factor analysis** |  |  |  |  |  |
| **KIDSCREEN-52** | **Ravens-Sieberer_2005_The KIDSCREEN-52 Quality of Life Measure for Children and Adolescents: Psychometric Results from a Cross-Cultural Survey in 13 European Countries** | **yes, cronbachs alpha (between .77 and .89)** |  |  |  |  | **yes, comparison to KINDLR (low to moderate correlation, high for scales physical and psychological wellbeing, school and mood and emotions), SES (lower SES, lower QOL, AGE (younger children higher QOL), gender (girls lower QOL), psychosomatic health (correlation lowto moderate)** |  |  |  | **yes, proportion of missing items (between1.37 and 2.85,** |
| **KIDSCREEN-52** | **Ravens-Sieberer_2008_The KIDSCREEN-52 Quality of Life Measure for Children and Adolescents: Psychometric Results from a Cross-Cultural Survey in 13 European Countries** | **yes, cronbachs alpha (.77-.89)** | **yest, test retest reliability with ICC (0.56 (Autonomy) to 0.77 (School Environment))** |  |  | **yes, confirmatory factor analysis** | **yes, comparison with PEDSQL (.44-.53), CHIP (.60-.56) YQOL (.56-.61), known groups (based on SDQ) SES, (lower SES, lower QoL)** |  |  |  |  |
| **KIDSCREEN-52** | **Robitail_2006_Validation of the European Proxy KIDSCREEN-52 Pilot Test Health- Related Quality of Life Questionnaire: First Results** | **yes, cronbachs alpha between .76 and .90** | **yes, intra-raterreliability with ICC (parent child).45 and .78)** |  |  | **yes, confirmatory factor analysis** | **yes, comparison with Qol measures (.relations ranged from 12 to .66;)convergent and divergent validity** |  |  |  |  |
| **KIDSCREEN-52** | **Shahabeddin-parizi_2014_Psychometric properties of KIDSCREEN health-related quality of life questionnaire in Iranian adolescents** | **yes, cronbachs alpha (between .6 and .94)** |  |  |  | **yes, confirmatory factor analysis** |  |  |  |  |  |
| **KIDSCREEN-52** | **Taliep_2012_Evaluating the construct validity of the KIDSCREEN-52 Quality of Life questionnaire within a South African context** | **yes, cronbachs alpha (.76 and .80)** |  |  |  | **yes, exploratory factor analysis** |  |  |  |  |  |
| **KIDSCREEN-52** | **Tzavara_2012_Reliability and validity of the KIDSCREEN-52 health-related quality of life questionnaire in a Greek adolescent population** | **yes, cronbachs alpha (between .73 and .90)** |  |  |  | **yes, confirmatory factor analysis** | **yes, comparison with SDQ (moderate to high correlation), known Groups (between chronic health conditions and a healthy sampe and SES (using Fas, low scores on fas low Qol)** |  |  |  |  |
| **KIDSCREEN-52, KIDSCREEN27** | **Ng_2015_Psychometric properties of the Chinese (Cantonese) versions of the KIDSCREEN health-related quality of life questionnaire** | **yes, cronbachs alpha (KS-52 between .84 and .95, KS-27 between .84 and .91)** | **yest, test retest reliability with ICC** |  |  | **yes, confirmatory factor analysis** | **yes, SES, SDQ (ks52 between .13 and .50, KS27 between .11 and .50), FAS (KS-52 between .05 and .27, ks-27 between .11 and .25)** |  |  |  |  |
| **KIDSCREEN-52, KIDSCREEN-27, KIDSCREEN-10** | **Baydur_2016_Reliability and validity study of the KIDSCREEN Health-Related Quality of Life Questionnaire in a Turkish child/adolescent population** | **yes, cronbachs alpha (Kidscreen-52 ranged from self .69 to .90 parent .68-.92, Kidscreen-27 self .78-.84 and parent .77-.81)** | **yest, test retest reliability(ICC K-52 self between .66 and .95, proxy .07 (financial resources) and .71, K-27 self between .74 and .94, K-10 self .81, parent .53))) and intra-rater (parent child) with ICC (k-52 between .43 (social acceptance) and .68 (physical well being)** |  |  | **yes, confirmatory factor analysis** | **yes, convergent-discriminant validity (KIDSCREEN AND KINDL (correlations moderate to high) and known groups (between children experiencing health problems, and healthy)** |  | **maybe? Comparison with KINDL** |  |  |
| **KIDSCREEN-52, KIDSCREEN-27, KIDSCREEN-10** | **Stevanovic_2013_Evaluating the Serbian version of the KIDSCREEN quality-of-life** | **yes, cronbachs alpha (self ks-52 between .58 (selfperception) and .88, Ks-27 between .78 and .83, ks-10 .80) (parent ks-52 between .63 (selfperception) and .88, Ks-27 between .70 and .83, ks-10 .76)** | **yes, intra-raterreliability with ICC (parent child) (ks-52 between .34 (mood and emotions) and .69, Ks-27 between .38 (social support and peers) and .63, and ks-10 .36)** |  |  |  | **yes, correlation with KINDLR (correlations between .45 and .65 on comparabe scales)** |  | **yes, correlation ks-27 with ks-52 between .72 and .98) (ks-10 with ks-27 between .55 and .8)** |  |  |
|  |  |  |  |  |  |  |  |  |  |  |  |

### Table 14. KINDL-R

| **Version** | **Reference and instrument identification** | **Internal consistency** | **Reliability** | **Measurement error** | **Content validity** | **Structural validity** | **Hypotheses testing** | **Cross-cultural validity** | **Criterion validity** | **Responsive-ness** | **feasibility** |
| --- | --- | --- | --- | --- | --- | --- | --- | --- | --- | --- | --- |
| **KINDL-R Serbian version (kid and kiddo)** | **Stevanovic_2009_Serbian KINDL questionnaire for quality of life assessments in healthy children and adolescents: reproducibility and construct validity** |  | **yes, test-retest with ICC (kid between .55 and .64, total sclae .84), Kiddo between .03 (school) and .75, total scale .8)** |  |  | **yes, exploratory factor analysis and confirmatory factor analysis** |  |  |  |  |  |
| **KINDL-R Serbian version** | **Stevanovic_2008_the psychometric study of the serbian kindl questionnaire for health rlated queality of life assessment in children and adolescents** | **yes, cronbachs alpha (KIDs between .46 and. 68, total scale .81 , Kiddo S between .45 and .72) total scle .83)** |  |  |  |  |  |  |  |  | **yes, with missing items** |
| **KINDL-R (norwegian version)** | **Helseth_2005_Assessing health-related quality of life in adolescents: some psychometric properties of the first Norwegian version of KINDL** | **yes, cronbachs alpha (between .53 and .82)** |  |  |  | **yes, exploratory factor analysis** |  |  |  |  |  |
| **KINDL-R (norwegian version)** | **Helseth_2006_Health-related quality of life in a Norwegian sample of healthy adolescents: Some psychometric properties of CHQ-CF87-N in relation to KINDL-N** |  |  |  |  |  |  |  |  |  |  |
| **KINDL-R (kids between 11 and 17)** | **Bullinger_2008_Psychometric properties of the KINDL-R questionnaire: results of the BELLA study** | **yes, cronbachs alpha (between .54 and .82)** |  |  |  |  | **yes, known groups (based on SDQ), comparison with KIDscreen (between .23 (selfpercention and .53) and corerlations between paren t and child ( all above .5 except self perception .23) discriminant validity; children with and without chronic disease. Significant differences for all subscales.** |  | **convergence validity, correlation with the KIDSCREEN-52** |  |  |
| **Kindl-R (farsi version** | **Rojhani_2016_Exploring the Psychometric Properties of the Farsi Version of Quality of Life Kindl Questionnaire for 4-7 Year-Old Children in Iran** | **yes, cronbachs alpha (between .34 (social relationship) and .74)** |  |  | **yes, experts reviewed the questionnaire)** |  | **yes, known groups (between healthy and ill child)** |  |  |  |  |
| **KINDL-R (age m 13.2 (2.1)** | **Meyer_2016_Health-related quality of life in children and adolescents: Current normative data, determinants and reliability on proxy-report** |  | **yes, agreement between parent and self (ICC between .56 (self esteem) and .78)** |  |  |  | **yes, age (QoL decreased with age), bmi (no relation found)** |  |  |  |  |
| **KIND-R (norwegian version) doen iets ingewikkelds misschien structural validity** | **Christophersen_2008_A Generalizability Study of the Norwegian Version of ${KINDL}^R$ in a Sample of HealthyAdolescents** |  |  |  |  |  |  |  |  |  |  |
| **Kiddy-KINDL-r** | **Villalonga_olives_2015_Self-reported health-related quality of life in kindergarten children: psychometric properties of the Kiddy-KINDL** | **yes, cronbachs alpha (between .10 (psychological well being) and .71, total scale .76)** | **yes, test-retest with ICC (total score. 83)** |  |  | **yes confirmatory factor analysis** | **yes, discriminant (difference for children with high performance in the social emotional scale and those with low performance) and dfiferences between boys and girls ( boys had lower score)** |  |  |  |  |
| **kiddo-KINDL-r (nepalese)** | **Yamaguchi_2010_Reliability and validity of a Nepalese version of the Kiddo-KINDL in adolescents** | **yes, cronbachs alpha (between .73 and. 84)** | **yes, test-retest with ICC (between .88 and .94)** |  |  |  | **yes, discriminant validity (between high and low scores on CES-D and gender)** |  |  | **yes, AUC, ROC curves** |  |
| **Kiddo-KINDL-R** | **Lee_2008_Cross-cultural Measurement Equivalence of the KINDL Questionnaire for Quality of Life Assessment in Children and Adolescents** | **yes, cronbachs alpha (between -.31 (school) and .81)** | **yes, test retest reliability (ranging from .43 to .77)** |  | **yes, focus group discussions** | **yes, exploratory factor analysis** | **yes, correlation with depression scale (correlations between -.59 and .50) and difference between boys and girls** |  |  |  |  |
| **Kiddo-KINDL-R** | **Pignatti TeixeiraI_2012_Cultural adaptation and validation of the KINDL questionnaire in Brazil for adolescents between 12 and 16 years of age** | **Yes, cronbachs alpha ranged from .12 (physical wellbeing) to .73)** |  |  |  | **yes,exploratory factor analysis** |  |  |  |  |  |
| **Kid-KINDL-R (chiniese version** | **Chan_2014_Psychometric Properties of the Chinese Version of the Kid-KINDLR Questionnaire for Measuring the Health-related Quality of Life of School-aged Children** | **yes, cronbachs alpha (child between .47 and. 77, parent between .55 and .79)** |  |  | **yes, expert panel** | **yes, principle components analysis** | **yes, known groups (healthy group and global delay group and comparison between age and sex (both not significant)** |  |  |  |  |
| **Kid-KINDL-R (chiniese version** | **Lee_2016_Psychometric evaluation and wording effects on the Chinese version of the parent-proxy Kid-KINDL** | **yes, cronbachs alpha (between .59 and . 86)** |  |  |  | **yes confirmatory factor analysis** | **yes, agreement between parent child (parents rated higher)** |  |  |  |  |
| **kid and kiddo** | **wee_2005_Validation of the English version of the KINDL' generic children's health-related quality of life instrument for an Asian population - results from a pilot test** | **yes, cronbachs alpha (kid between .11 (social) and .72, total .75), Kiddo between .31 (school) and. 75, total .84)** |  |  | **yes, face validity** |  | **yes, discriminant validity (difference between patients and controls)** |  |  |  |  |
| **kid and kiddo** | **wee_2007_Factor structure of the Singapore English version of the KINDL® children quality of life questionnaire** | **yes, cronbachs alpha kid between .46 and .71, total .79), Kiddo between .44 (school) and. 84, total .83)** |  |  |  | **yes, factor analysis** |  |  |  |  |  |
|  | **Erhart_2009_Measuring adolescents' HRQoL via self reports and parent proxy** | **yes, cronbachs alpha (self between .53 and .82, parent between .62 and .86)** | **yes, intrarater (proxy self) with ICC (between .24 and .45)** |  |  | **yes, confirmatory factor analysis** | **yes, known groups (children with and without special health care needs), and correlation with SDQ scales (between. 33 and .49)** |  | **yes convergence validity between proxy and self version** |  |  |
|  | **Jafari_2014_Measurement Equivalence of the KINDL Questionnaire Across Child Self-reports and Parent Proxy-reports: A Comparison Between Item Response Theory and Ordinal Logistic Regression** |  |  |  |  |  | **yes, relation between parent and self based on IRT with dif. Overal 50% agreement** |  |  |  |  |
|  | **Ravens_siebereer_2008_Health-related quality of life in children and adolescents in Germany: results of the BELLA study** | **yes, cronbachs alpha (parent between .64 and .74, self 7 tot 11 years (.84) 11-17 years(.87)** |  |  |  | **yes, structural validity assed with the MAP program using campbells multitrait approach** | **yes, known groups (chronic pain, astma , mental health (based on SDQ), sex (no differences)** |  |  |  |  |

Note. The KINDL-R was developed by Ravens-Sieberer, U. & Bullinger, M. (1998a). Assessing health related quality of life in chronically ill children with the German KINDL: first psychometric and content-analytical results. Quality of Life Research, Vol. 4, No 7; & Ravens-Sieberer, U. & Bullinger, M. (1998b). News from the KINDL-Questionnaire – A new version for adolescents. Quality of Life Research, 7, 653.

### Tabel 15. Multidimensional students life satisfaction scale

| **Version** | **Reference and instrument identification** | **Internal consistency** | **Reliability** | **Measurement error** | **Content validity** | **Structural validity** | **Hypotheses testing** | **Cross-cultural validity** | **Criterion validity** | **Responsive-ness** | **feasibility** |
| --- | --- | --- | --- | --- | --- | --- | --- | --- | --- | --- | --- |
| **Multidimensional Student's Life Satisfaction Scale (MSLSS)** | **Huebner (1994)** | **Yes cronbachs alpha between .79 and .83, total scale .92** |  |  |  | **yes, factor analysis** | **yes difference between gender, age, race, and correlation with LSDS, QSLS** |  |  |  |  |
| **Multidimensional Student's Life Satisfaction Scale (MSLSS)** | **Greenspoon & Saklofske (1997)** | **Yes between .72 and .90** |  |  |  | **yes, factor analysis** | **yes correlation between diffent quesionnaires** |  |  |  |  |
| **Multidimensional Student's Life Satisfaction Scale (MSLSS)** | **Huebner & Gilman (2002)** | **yes, between .72 and .92** |  |  |  | **yes, exploratory factor analysis** | **yes, difference between age, gender** |  |  |  |  |
| **Multidimensional Student's Life Satisfaction Scale (MSLSS)** | **Haranin 2007** | **Yes, cronbachs alphe between .77 and .87** | **Cronbach's alpha: 0,75 (p. 620)** | **no** | **no** | **no** | **concurrent, predictive, incremental** | **no** |  |  |  |
| **Brief Multidimensional Student's Life Satisfaction Scale (BMSLSS)** | **Huebner 2011** | **yes, cronbachs alpha .76** | **yes, test-retest reliability coeficient .52** | **no** | **no** |  | **Yes, predictive validity** |  |  |  |  |
| **Brief Multidimensional Student's Life Satisfaction Scale (BMSLSS)** | **Athay 2012** | **Ye scronbachs alpha . 77** |  | **Yes, standard error of measurement (SEM=.40)** |  | **yes confirmatory factor analysis** | **discriminant, constructcomparison with youth life satisfaction** |  | **concurrent, predictive (Huebner et al. p.165)** | **yes** |  |
| **Brief Multidimensional Student's Life Satisfaction Scale (BMSLSS)** | **Hashim & Areepattamannil (2017)** | **yes, cronbachs alpha = .82** | **test-retest reliability coefficient of the BMSLSS was 0.94** |  |  | **yes, confirmatory factor analysis** | **yes, conparison with life satisfaction** |  |  |  |  |
| **Brief Multidimensional Student's Life Satisfaction Scale (BMSLSS)** | **Zeng et al. (2017)** |  |  |  |  | **yes, confirmatory factor analysis** |  |  |  |  |  |

Note.

### Tablel16. PEDSQL

| **Reference and instrument identification** | **Internal consistency** | **Reliability** | **Measurement error** | **Content validity** | **Structural validity** | **Hypotheses testing** | **Cross-cultural validity** | **Criterion validity** | **Responsive-ness** | | **feasibility** | |
| --- | --- | --- | --- | --- | --- | --- | --- | --- | --- | --- | --- | --- |
| ainuddin et al. 2013_Psychometric properties of the self-report Malay version of the Pediatric Quality of Life (PedsQLTM) 4.0 Generic Core Scales among multiethnic Malaysian adolescents | | Cronbach’s a  values ranging from .70 to .89 |  |  |  | yes, exploratory factor analysis | Yes, differences in Qol among age, gender and race. Older adolescents better QoL, Males better emotional functioning, indian adolescents better overall QoL than Malay. |  |  | |  | |
| Amiri_2010_ Reliability and validity of the Iranian version of the Pediatrie Quality of Life Inventory™4.0 Generic Core Scales in adolescents | | yes cronbacks a values ranging from ..68-.88 for the adolescent version and .67-.89 for the proxy version | yes, intrarater (parent vs self) reliability with ICC (.82, 95%CI.79-.84) |  |  | yes, exploratory and confirmatory factor analysis | yes, relation between parent and proxy (ranging from .53-.65) construct validity (healthy scored higher than chronically ill) |  |  | | yes, number of missing items, no floor effects, ceiling effects 1.5%. | |
| arabiat_2011_Cross-cultural Validation of the Pediatric Quality of Life Inventory 4.0 (PedsQL ) generic core scale into Arabic Language | | yes , cronbachs alpha self ranging from .66-.91 for self, for parent proxy (.62-.89) |  |  |  |  | yes discriminant validity (chronically ill, cancer, controls), relation between parent and self ranging from 1.0 (physical)- .429(social) |  |  | |  | |
| atilola_2013_PedsQLTM 4.0 Generic Core Scales for adolescents in the Yoruba language: Translation and general psychometric properties | | yes, cronbachs alpha (.71-.91) |  |  | yes, parents/adolescents and expert panel | yes, confirmatory factor analysis | yes construct validity (mental or psysical health problems/controls) and agreement between SDQ and PEDSQL (correlations ranging from -.22-.08) |  | yes | |  | |
| Bastiaansen_2004_Measuring Quality of Life in Children Referred for Psychiatric Problems: PsychometricProperties of the PedsQL™ 4.0 Generic Core Scales | | yes cronbachs alpha 6-7 years (.40-.63), 8-12 (.63-.85), 13-18 (.57-.87), parent all ages (.69-.87) | yes,interparent agreement with ICC (.86-.91) |  |  | yes, confirmatory factor analysis | yes, convergent validity (CBCL en PEDSQL (child -.24, parent -.62) and discriminant validity (IQ) (.09) |  | yes, agreement between patients and healthy | |  | |
| buck_2012_The PedsQL™ as a measure of parent-rated quality of life in healthy UK toddlers: Psychometric properties and cross-cultural comparisons | | yes, cronbachs alpha (ranging from .58-.82) |  |  |  |  | Correlation with age(r=.003-.031) and gender |  |  | | Yes, missing items (.8-1/2%), floor (0%) and ceiling effects(8-57%) | |
| chan_2005_Preliminary validation of the Chinese version of the Pediatric Quality of Life Inventory | | yes, chronbachs alpha (all above >.7) except in emotional (.62) and school functioning (.55) | yes, test retest reliability (ICC ranging from .62-.81) |  | yes, face to face interviewing |  | yes, construct validity (disabled vs healthy) and agreement between parent and child (total score r =.78) |  |  | |  | |
| chen_2007_Reliability and Validity of the Pediatric Quality of Life Inventory™ (PedsQL™) ShortForm 15 Generic Core Scales in Japan | | yes, chronbachs alpha (self .71-.79), parent .81-.86) | no ICC, dus niet gescoord. |  |  | yes, factor analysis with oblique ratation. | yes, contruct validity (headache, abdominal pain vs healthy), gender differences and agreement between parent and child |  |  | | yes, number of missing items(0-1.6%), floor (0-.9%) and ceiling effects (2.4-52.4) | |
| amaias arias_2017_Construct and Criterion Validity of the PedsQL™ 4.0 Instrument (Pediatric Quality of Life Inventory) in Colombia | |  |  |  |  | yes, exploratory factor analysis and confirmatory | yes agreement between kidscreen and PedsQL (spearman rho between .12-.48) |  |  | |  | |
| danansuriya_2012_Psychometric properties of the Sinhala version of the PedsQL™ 4.0 Generic Core Scales in early adolescents in Sri Lanka | | yes, chronbachs alpha (self between .60-.85(total scale) and parent (.67-.86(totalscale)) | no ICC, dus niet gescoord. |  | yes, expert panel |  | yes, (between asthma and healthy) and parent en self (rho between .18(school) and .44 (physical functioning) |  |  | | yes, missing items(.39%), and completion rates (100%) | |
| desai_2014_Validity and Responsiveness of the Pediatric Quality of Life Inventory (PedsQL) 4.0 Generic Core Scales in the Pediatric Inpatient Setting | |  |  |  |  |  | yes, between groups with differing medical severity and predictive validity |  |  | |  | |
| engelen_2009_Health related quality of life of Dutch children: psychometric properties of the PedsQL in the Netherlands | | yes chronbachs alpha (.53-.85) |  |  |  |  | yes, construct validity (healthy vs chronically ill) and differences with age (younger higher scores), gender (boys higher in emotional functioning) |  |  | |  | |
| fereirra_2014_Reliability and validity of PedsQL for Portuguese children aged 5–7 and 8–12 years | | yes, cronbachs alpha (5 to 7 years old (.36-.78) parents (.74-.92), children 8-12(.62-.89) parents (.64-.89) | yes, test retest within 72 hours (5 to 7 years old (.80-.97 parents (.97-.99), children 8-12(..93-.98) parents (.90-.99) |  |  |  | yes, construct validity (healthy vs chronically ill) and parent and child (r tussen .26 (rmotional functioning) and .76 physical functioning) and relation with KINDL-R (total score between .51 and .64) |  |  | |  | |
| gheissari_2012_Validation of Persian Version of PedsQL™ 4.0™ Generic Core Scales in Toddlers and Children | | yes cronbachs alpha (.73 and .9) | Yes, ICC between parent and child (agreement excellent for social and school functioning, good agreement for total score and psychial functioning and low agreement for the emotional functioning (ICC.32) |  | yes face validity and validation by physicians with kappa rating | yes exploratory factor analysis | yes, construct validity (healthy vs chronically ill) and parent child agreement |  |  | | yes, missing data(3.6 in children, 2.2 in parents), floor(no) and ceiling effects( between 4 and 10%) | |
| hao_2010_Psychometric properties of the Chinese version of the Pediatric Quality of LifeInventory™ 4.0 generic core scales | | Yes, cronbachs Alpha between .80 and .95) | yes, ICC, parent child agreement ( ranging from .64-.78) |  |  | yes, confirmatory factor analysis | yes, construct validity (healthy vs 5 ill groups) and item scale correlation |  |  | | yes, response rate (95%) missing items (1.33%) | |
| hee_2008_Validation of the Korean version of the pediatric quality of life inventory™ 4.0 (PedsQL™) generic core scales in school children and adolescents using the rasch model | | Yes, cronbachs alpha (self between .72-.90) proxy (.75-.90) | yes ICC (parent child(. 47-.59)) |  |  | yes, confirmatory factor analysis, and rasch method | yes, construct validity (healthy vs chronically ill), |  |  | | yes.missing values (1.7%) | |
| huguet_@008_Development and Psychometric Evaluation of a Catalan Self- and Interviewer-Administered Version of the Pediatric Quality of Life InventoryTM Version 4.0 | | yes, cronbachs alpha between .60 and .77) |  |  |  | yes, confirmatory factor analysis | yes, constrruct validity (children with a medical diagnosis vs health children) and comparison with KINDL(total score(.36) |  |  | | yes, missing values(.24% ) no floor effects and (49.1% ceiling for physical health) | |
| Jafari_2011_Health-related quality of life of Iranian children with attention deficit/hyperactivitydisorder | | yes Cronbachs alpha self (between .72 and .86) and parent (.71 and .82) |  |  |  | yes, exploratory factor analysis | yes, known group (children with ADHD, compard to school children) |  |  | | yes, missing items | |
| Jafari_2012_Using Rasch rating scale model to reassess the psychometric properties of the Persian version of the PedsQLTM 4.0 Generic Core Scales in school children | | yes cronbachs alpha (self between .70 and .85, parent between.70 and .89) |  |  |  | yes,Confirmatory factor analysisen rasch |  |  |  | |  | |
| Kaartina_2015_Adolescent self-report and parent proxy-report of health-related quality of life: an analysis of validity and reliability of PedsQL™ 4.0 among a sample of Malaysian adolescents and their parents | | yes cronbachs alpha (self between .75 and .89 and parent between .72 and .94) |  |  |  | yes, confirmatory factor analysis | Yes, comparison between parent als self. Gender (male higher emotional and social) association with BMI |  |  | |  | |
| Klatchoian_2008_Quality of life of children and adolescents from São Paulo: reliability and validity of the Brazilian version of the Pediatric Quality of Life InventoryTM version 4.0 Generic Core Scales | | yes, cronbachs alpha self between .60 and .88, parent between .62 and .88) |  |  |  |  | yes, known group (juvinile atritis and health) parent and child relation (exact not reported. Correlations were significant with highest correlation in physical functioning) , VAS scale (-.36), CHAQ (..62) and CHQ Physical (.59, psychosocial (.65) |  |  | |  | |
| Kobayashi_2010_Measuring quality of life in Japanese children: Development of the Japanese version of PedsQL | | Yes, cronbachs alpha (child toddler self .28- and .64, parent .71-.90, Schoolchild (.52 and .85, parent .74 and .92) | yes, retest reliability with ICC .88 child, parent. 86) |  | yes, expert panel |  | yes, known groups (chronic needs, mental condistion, combination and healthy) with parent (r tussen .15 and .40) and depression scale (t between -.76 and ..38), and illness |  |  | | yes, missing items (self report .81%, parent .66%) | |
| Laaksonen_2007_Paediatric health-related quality of life instrument for primary school children: cross-cultural validation | | yes, cronbachs alpha child.73-.89, parent .69-.89) |  |  | yes, cognitive interview |  |  |  |  | |  | |
| Limbers_2008_Factorial Invariance of Child Self-Report across Age Subgroups: A Confirmatory Factor Analysis of Ages 5 to 16Years Utilizing the PedsQL 4.0 Generic Core Scales | |  |  |  |  | yes, factorial analysis |  |  |  | |  | |
| Limbers_2011_patient reported pediatric quality of life inventory4.0. generic core sclaes in pediatric patients with ADHD | | yes, cronbachs alpha (all apha above.70 except school functioning patient self report (.65) | yes, self vs parent ICC (between .13 and .35) |  |  |  | yes, known groups (ADHD, cancer, Healthy) and correlation with ADHD sclaes (-.16), number of diagnosis (-.27) duration mediation(-.21) and months since diagnosis (-/20) |  |  | | yes, missing items (between.2 and.5%) | |
| Lin_2013_Measurement Equivalence across Child Self-Reports and Parent-Proxy Reports in the Chinese Version of the Pediatric Quality of Life Inventory Version 4.0 | |  | yes, test retest reliabilityparent child agreement (all between .77 and .90) |  |  | yes, confirmatory factor analysis | yes, differences parent child |  |  | |  | |
| Lin_2012_Psychometric properties and gender invariance of the Chinese version of the self-reportpédiatrie quality of life inventory version 4.0: short form is acceptable | | yes, cronbachs alpha (between .68-.90 long form, .62 and.87 short form | yes, test retest, ICC |  |  | yes, confirmatory factor analysis | yes, between depression scale and pedsql |  |  | |  | |
| Newman et al. 2010_Factorial Invariance of Child Self-Report Across English and Spanish Language Groups in a Hispanic Population Utilizing the PedsQL™ 4.0 Generic Core Scales | |  |  |  |  | yes, confirmatory factor analysis enz. |  |  |  | |  | |
| Pakpour_2013_Psychometric properties of the Iranian version of the Pediatric Quality of Life Inventory™ Short Form 15 Generic Core Scales | | yes, cronbachs alpha (self between .72 and .82, parent between .75 and .86) | yes, test retest reliability ICC (between .70 and .79 self, and parent (.72 and .81), parent child agreement ICC (between .29 and .59) |  |  | yes, confirmatory factor analysis | yes, known groups (school children vs paediatric patients) |  |  | | yes, missing items, floor (between .4 and6.1%) and ceiling effects (between 1.3 and 38%) | |
| Petersen_2009_Psychometric properties of the Swedish PedsQL, Pediatric Quality of Life Inventory 4.0 generic core scales | | yes, cronbachs alpha (full form between .71 and .90, short form (netwee .63 and .86) | yes, inter, en intraraterreliability, ICC interrater reliability (long: .38-.56) long(.35-.56), intrarater ( short(.84-.91) long(.83-.90) |  |  | yes, confirmatory factor analysis | yes, parent child ratings with kappo above .40convergent validity between cbcl (-.56 tot -.60)and pedsql |  |  | |  | |
| reinfjell_2006_Measuring health-related quality of life in young adolescents: Reliability and validity in the Norwegian version of the Pediatric Quality of Life Inventory™ 4.0 (PedsQL) generic core scales | | yes, cronbachs alpha (self between .73 and .84) parent between .75 and .88) | yes, ICC, parent child agreement (all <.40) |  |  | yes, exploratory factor analysis | Yes agreement between parent and child (between .22 and .35) and gender differences, girls scoring lower than boys |  |  | |  | |
| Sritipsukho_2013_Reliability and validity of the Thai version of the Pediatric Quality of Life Inventory 4.0 | | yes, cronbachs alpha (self between .68 and .76), parent between .69 and .88) | yes, retest reliability with ICC (self .66-.73) (parent .65-.71) |  |  |  | yes, known groep validity (healthy children vs chronic health conditions. agreement between parent and child |  |  | |  | |
| Stevanović _2011_Some psychometric properties of the Pediatric Quality of Life Inventory™ Version 4·0Generic Core Scales (PedsQL™) in the general Serbian population | | yes, cronbachs alpha between (.69 and .86) |  |  |  | yes, confirmatory factor analysis | yes, agreement between sdq and pedsql (between -.42 and .16 for correpsonding scales) |  |  | |  | |
| Ozden uneri_2008_Validity and Reliability of Pediatric Quality of Life Inventory for 2- to 4- Year-Old and 5-to 7-Year-Old Turkish Children | | yes, cronbachs alpha (self between .80 and .86, parent between .67 and .86) |  |  |  |  | yes, difference between healthy and children with acure diseases. And parent child agreement (low correlations), boys scoring lower than girls |  |  | |  | |
| Uneri_2007_The Validity and Reliability of the Turkish Pediatric Quality of Life Inventory for Children 13-18 Years Old | | yes, cronbachs alpha (self between .60 and .82, parent between .711 and .877) |  |  |  |  | yes, difference between healthy, accute and chronic disease, and parent and child (r between. .37 (docial functioning) and .82 (psychosocial health total score) |  |  | |  | |
| Upton_2005_Measurement properties of the UK-English version of the Pediatric Quality of Life Inventory™ 4.0 (PedsQL™) generic core scales | | yes, cronbachs alpha (all scores >.70 tot score exceeded .90) |  |  |  |  | yes, comparison of health and chronic health conditions and agreement between parent and child, gender differences |  |  | |  | |
| Varni_1999)The PedsQL™: Measurement Model for the Pediatric Quality of Life Inventory | | yes, cronbachs alpha (between .67 and .83) |  |  |  | yes factor analysis | yes, comparison with several questionnaires, and patient group |  |  | | yes, percentage missing items (less than .1%) | |
| Varni_2001_PedsQL™ 4.0: Reliability and Validity of the Pediatric Quality of Life Inventory™ Version4.0 Generic Core Scales in Healthy and Patient Populations | | yes, cronbachs alpha (self between .68 and .88, parent between.75 and .90) |  |  |  | yes, factor structure analysed via MTMM analysis of the subscales and factor analysis of the items. | yes, know groups (healthy vs chronic ill, accutely ill), relation with needed care, days misse from work. |  |  | | yes, percentage missing items ( between 1.54 and 1.95%) | |
| Varni_2006_The PedsQL™ as a patient-reported outcome in children and adolescents with Attention-Deficit/Hyperactivity Disorder: a population-based study | | yes, cronbachs alpha self between .83 and .92, parent between .76 and .92) |  |  |  |  | Differences between ADHD, Cancer, CP and Healthy, Parent child agreement (between .59 (schoool) and .69 (psychosocial health) | yes, with adhd symptoms and know groups and parent child agreement |  | | yes, percentage missing items (self 0% parent 4.9%) | |
| Varni_2008_Factorial Invariance of the PedsQL™ 4.0 Generic Core Scales Child Self-Report Across Gender: A Multigroup Confirmatory Factor Analysis with 11,356 Children Ages 5 to 18 | |  |  |  |  | yes, confirmatory factor analysis |  |  |  | |  | |
| Varni_2008_Longitudinal Factorial Invariance of the PedsQL™ 4.0 Generic Core Scales Child Self-ReportVersion: One Year Prospective Evidence from the California State Children's HealthInsurance Program (SCHIP) | |  |  |  |  | yes, confirmatory factor analysis |  |  |  | |  | |
| Gkoltsiou_2008_Measuring Health-Related Quality of Life in Greek Children: Psychometric Properties ofthe Greek Version of the Pediatric Quality of Life Inventory™ 4.0 Generic Core Scales | | yes, cronbachs alpha (self between .65 and .83, parent between .71 and .84) | yes, test retest (all ICCs above. 60) and intraclassreliability with ICC (between .61 and .88) |  |  | yes, principal conponent analysis | yes, comparison between self and proxy (correlation moderate to high r .22 -.29) and chronically ill and healthy children. |  |  | |  | |
| Varni_2011_The PedsQL™ Infant Scales: feasibility, internal consistency reliability, and validity inhealthy and ill infants | | yes, cronbachs alpha (between .72 and .90) |  |  |  | yes, confirmatory factor analysis | yes, discriminant analysis( known groups, chronically ill, accutely ill and healthy) |  |  | | yes, percentage missing items (.7%) | |
| Varni_2003_The PedsQLy* 4.0 as a Pediatric Population Health Measure: Feasibility, Reliability, and Validity | | yes, cronbachs alpha ( child between .71 and .87, parent between .74 and .88) |  |  |  |  | yes, between healthy and chronic health, concordance child and parent (higher correlations with increasing age, and SF-36, gender age, language, race |  |  | | yes, percentage of missing items (self 1.8% parent 2.4%) | |
| Varni_2007_How young can children reliably and validly self-report their health-related quality of life?: An analysis of 8,591 children across age subgroups with the PedsQL™ 4.0 Generic Core Scales | | Yes cronbachs alpha between .70 and .87 for different age grou[s | yes, parent child agreement, ICC |  |  |  | yes, between healthy and chronic health |  |  | | yes, percentage missing items (1.2%) | |
| Varni_2007_Parent proxy-report of their children's health-related quality of life: an analysis of 13,878 parents' reliability and validity across age subgroups using the PedsQL™ 4.0 Generic Core Scales | | yes, cronbachs alpha | yes, parent child agreement, ICC (between .44 an .70) |  |  |  | yes, known groups (between chronic health conditions and healty) |  |  | | yes, missing items | |
| Viecili_2015_Reliability and Validity of the Pediatric Quality of Life Inventory With Individuals With Intellectual and Developmental Disabilities | | yes, cronbachs alpha (between. 78 and .90) |  |  |  | yes, exploratory factor analysis | yes, known groups, IDD+ASD compared to ASD, convergent validity thrhough comparison with SDQ (between -.70 and .27 |  |  | | yes, missing items (less than 1%) | |
| Health-related quality of life of Estonian adolescents: reliability and validity of the PedsQLTM 4.0 Generic Core Scales in Estonia | | yes, cronbachs alpha between .50 and .81 |  |  |  | yes, confirmatory factor analysis | Yes, gender differences. |  |  | |  | |
| Limbers_2008_Factorial invariance of child self-report across socioeconomic status groups: a multigroup confirmatory factor analysis utilizing the PedsQL 4.0 Generic Core Scales | |  |  |  |  | yes, confirmatory factor analysis | Yes diffences with SES |  |  | |  | |
| Limbers_2008_Factorial invariance of child self-report across healthy and chronic health condition groups: A confirmatory factor analysis utilizing the PedsQL™ 4.0 Generic Core Scales | |  |  |  |  | yes, confirmatory factor analysis | Yes, known groups (chronic health condition and healthy) |  |  | |  | |

NOTE. The pedsql was developed by Varni, J.W., Seid, M., & Rode, C.A. (1999). The PedsQLTM: Measurement model for the Pediatric Quality of Life InventoryTM. Medical Care, 37, 126-139.

### Tabel 17. QOLPAV

| **Version** | **Reference and instrument identification** | **Internal consistency** | **Reliability** | **Measurement error** | **Content validity** | **Structural validity** | **Hypotheses testing** | **Cross-cultural validity** | **Criterion validity** | **Responsive-ness** | **feasibility** |
| --- | --- | --- | --- | --- | --- | --- | --- | --- | --- | --- | --- |
| **Quality of Live Profile: Adolescent Version (QOLPAV)** | **Rapheal et al (1996)** | **yes, cronbachs alpha between .67 and .87** |  |  | **yes, adolesence rate each item for relevance** | **yes, factor analysis** | **yes, discriminant validity** |  |  |  |  |
| **Quality of Live Profile: Adolescent Version (QOLPAV)** | **Bradford et al (2002)** | **yes, cronbachs alphe between .65 and .94** |  |  |  | **yes, factor analysis** | **yes, discriminant validity (between age, gender, ethnicity, parental occupation and number of siblings)** |  |  |  |  |
| **Quality of Live Profile: Adolescent Version (QOLPAV)** | **Meuleners et al (2003)** |  |  |  |  | **yes, confirmatory factor analyses and SEM** |  |  |  |  |  |
| **Quality of Live Profile: Adolescent Version (QOLPAV)** | **Meuleners et al (2005)** |  |  | **no** |  | **yes, confirmatory factor analyses and SEM** |  | **no** | **no** | **no** |  |

Note.

### Tabel 18. Quality of well being scale – mental health subscale

| **Reference and instrument identification** | **Internal consistency** | **Reliability** | **Measurement error** | **Content validity** | **Structural validity** | **Hypotheses testing** | **Cross-cultural validity** | **Criterion validity** | **Responsive-ness** | **feasibility** |
| --- | --- | --- | --- | --- | --- | --- | --- | --- | --- | --- |
| Sarkin et al. (2013) | Cronbach's alpha: 0,827 in sample 1 and . 842 in sample 2 | ICC between raters of which scales to include: 0,77 (p. 1686) | no | no | no | yes, agreement with other scales )sf-36 (r between -.663 and -.72), EQ-5D (-.61), HUI (between -.591 and -.631) and POMS ( .77) | no |  | no |  |

### Tabel 19. TNO-AZL child quality of life (TACQOL)

| **Reference and instrument identification** | **Internal consistency** | **Reliability** | **Measurement error** | **Content validity** | **Structural validity** | **Hypotheses testing** | **Cross-cultural validity** | **Criterion validity** | **Responsive-ness** | **feasibility** |
| --- | --- | --- | --- | --- | --- | --- | --- | --- | --- | --- |
| **Verrips et al (1999)** | **Cronbachs alphe between .65 and .84** | **ICC between parent and child between .44 and .61** |  |  |  | **Pearson correlation with KINDL: 0,24-0,6 (Verrips et al. p. 190), and differences between children with chronic illness and who had undergone medical treatment** |  |  |  |  |
| **Vogels et al (1998)** | **Cronbach's alpha: between .59 and .89** | **Test retest reliability between .04 and .38** | **no** | **no** | **no** | **no** | **no** |  | **no** |  |

Note.

### Table 20. TAPQOL

| **Reference and instrument identification** | **Internal consistency** | **Reliability** | **Measurement error** | **Content validity** | **Structural validity** | **Hypotheses testing** | **Cross-cultural validity** | **Criterion validity** | **Responsive-ness** | **feasibility** |
| --- | --- | --- | --- | --- | --- | --- | --- | --- | --- | --- |
| **Bunge_2005_Reliability and validity of health status measurement by the TAPQOL** | **yes, cronbachs alpha, between .46 and .97** | **yes, test-retest with ICC(between .35 and .88 for the total group)** |  |  |  | **yes, discriminant analysis (known groups)** |  |  |  | **yes, response rate (83%) and missing items (circa 1% per item)** |
| **Fekkes_2000_Development and Psychometric Evaluation of the TAPQOL: A Health-Related Quality ofLife Instrument for 1-5-Year-Old Children** | **yes, cronbachs alpha (Sample 1 preterm babies 0.66 to 0.88, Sample 2 well-baby clinic .43 and .84)** |  |  |  | **yes, principal component analysis** | **yes known groups, and comparison with FS-II scores** |  |  |  | **yes** |
| **Rajmil_2011_Reliability and validity of the Spanish version of the TAPQOL: A health-related quality of life (HRQOL) instrument for 1- to 5-year-old children** | **yes, cronbachs alpha (between.57 and .91)** |  |  |  | **yes, principal component analysis** | **yes, known groups (group of healthy children, premature children, and children consulting at the respiratory unit (difference between age groups)** |  |  |  | **yes, response rate (95%)** |
| **Schepers_2017_Health related quality of life in Dutch infants, toddlers, and young children** | **yes, cronbachs alpha .60–.92** |  |  |  |  | **yes, known groups (chronic health condition compared to healthy) and difference with age and gender** |  |  |  |  |
| **Tay_2015_Cross-cultural adaptation and validation of the Malay language version of the TZO-AZL Preschool Children Quality of Life questionnaire: A health-related quality of life instrument for preschool children** | **yes, cronbachs alpha .69 to .90.** |  |  |  | **yes, principal component analysis** | **yes, known groups (preterm vs term born children, children with good and less good health and children with chronic conditions and no contditions)** |  |  |  |  |
| **vanAgt_2005_Quality of Life of Children with Language Delays** | **yes, cronbachs alpha (between .63 and .82)** |  |  |  | **yes,factor analysis** | **yes, agreement with questionnaire for langueage delays (between.02 (anxiety and .61 higher correlations in the group with language disorders) en known groups (with language disorders and without), and ROC curve** |  |  |  | **yes, number of missing values (1.9-6.7%)** |
| **Verrips_2000_Measuring health-related quality of life in adolescents: agreement between raters and between methods of administration** | **yes, cronbachs alpha between .55 and .80 for all administration methods)** | **yes with ICC (interrater) (between .27 and .72 for all administration methods), also looked at inter-method agreement** |  |  |  |  |  |  |  |  |

Note. TNO. TNO-AZL pre-school children quality of life users manual. TNO PG, Leiden, Netherlands, 2004.

### Table 21. Youth quality of life instrument (YQOL)

| **Reference and instrument identification** | **Internal consistency** | **Reliability** | **Measurement error** | **Content validity** | **Structural validity** | **Hypotheses testing** | **Cross-cultural validity** | **Criterion validity** | **Responsive-ness** | **feasibility** |
| --- | --- | --- | --- | --- | --- | --- | --- | --- | --- | --- |
| **Patrick et al (2002)** | **Cronbach's alpha: 0,77-0,96 (Patrick et al. p.296)** | **test-retest ICC: between .74 and .85 (Patrick et al. p.296)** | **no** | **yes** | **no** | **construct/discriminant validity (comparison with CDI ( r=-.58) with the FDI ( -.26) comparison to KINDL; correlation 0,73 (Patrick et al. p.296), between high depressive and high ADHD, and self reported disability** | **no** |  | **no** |  |
| **Jiang et al. (2014)** | **Cronbach's alpha: 0,84-0,96** | **test-retest ICC: >0,7** | **Standard error of measurement 8,4-12,1** |  |  | **construct/discriminant validity (comparison with PedsQL's comparable dimensions, r=0,21-0,53), known groups (significant differences across weight status, age and genders)** |  |  |  |  |

Note.

# Appendix 8. Domains of QoL per age group

### Table Domains of QoL 0-8 years

| Questionnaire | Age range | Physical health | Psychological state | level of independence | social relationships | Personal beliefs | relation to salient features of environment |
| --- | --- | --- | --- | --- | --- | --- | --- |
| Chip-CE | 6-11 | x | x |  | x | x | x |
| CHQ-PF50,28 | 5-18 | x | x |  | x | x | x |
| DUX-25- parent form | 6-12 | x | x |  | x |  | x |
| KINDL | 4-18 | x | x |  | x | x | x |
| PEDSQL | 5-17 | x | x |  | x |  |  |
| TACQOL-parent | 6-12 | x | x | x |  |  |  |
| HUI2/3 | 5+ | x | x |  |  |  |  |
| ITQOL | 2 months to 5 years | x | x |  | x |  | x |
| CHUd9 | 7-17 | x | x | x |  |  |  |
| CHSCH | 2-5 | x | x | x |  |  |  |
| GCQ | 6-16 |  |  |  |  |  |  |
| QWB |  |  |  |  |  |  |  |

### Table Domains of QoL 9-12 years

| Questionnaire | Age range | Physical health | Psychological state | level of independence | social relationships | Personal beliefs | relation to salient features of environment |
| --- | --- | --- | --- | --- | --- | --- | --- |
| Chip-CE | 6-11 | x | x |  | x | x | x |
| CHQ-CF87 | 10-18 | x | x |  | x | x | x |
| CHQ-PF50,28 | 5-18 | x | x |  | x | x | x |
| DUX-25- parent form | 6-12 | x | x |  | x |  |  |
| DUX-25- Child form | 8-12 | x | x |  | x |  |  |
| KINDL | 4-18 | x | x |  | x | x | x |
| KINDLR Child &parent | 8-16 | x | x |  | x | x | x |
| PEDSQL | 5-17 | x | x |  | x |  |  |
| TACQOL-parent | 6-12 | x | x | x |  |  |  |
| TACQOL-child | 8-12 | x | x | x |  |  |  |
| HUI2/3 | 5+ | x | x |  |  |  |  |
| EQ-5D-Y | 8+ | x | x | x |  |  |  |
| MSLSS | 8-18 |  |  |  | x | x | x |
| Kidscreen | 8-18 | x | x | x | x |  | x |
| CHUd9 | 7-17 | x | x | x |  |  |  |
| 17D | 8-11 | x | x | x | x | x |  |
| CQOL | 9-15 | x | x | x | x | x | x |
| GCQ | 6-16 |  |  |  |  |  |  |
| QWB |  |  |  |  |  |  |  |

### Table Domains of QoL 13-18 years

| Questionnaire | Age range | Physical health | Psychological state | level of independence | social relationships | Personal beliefs | relation to salient features of environment |
| --- | --- | --- | --- | --- | --- | --- | --- |
| Chip-AE | 11-17 | x | x |  | x | x | x |
| CHQ-CF87 | 10-18 | x | x |  | x | x | x |
| CHQ-PF50,28 | 5-18 | x | x |  | x | x | x |
| KINDLR | 4-18 | x | x |  | x | x | x |
| KINDLR Child &parent | 8-16 | x | x |  | x | x | x |
| PEDSQL | 5-17 | x | x |  | x |  |  |
| YQOL | 12-18 |  |  |  | x | x | x |
| HUI2/3 | 5+ | x | x |  |  |  |  |
| AQOL | 12-18 |  |  |  | x | x | x |
| EQ-5D-Y | 8+ | x | x | x |  |  |  |
| MSLSS | 8-18 |  |  |  | x | x | x |
| QOLPAV | 14-20 | x | x |  | x | x | x |
| Kidscreen | 8-18 | x | x | x | x |  | x |
| CHUd9 | 7-17 | x | x | x |  |  |  |
| 16D | 12-15 | x | x | x | x | x | x |
| CQOL | 9-15 | x | x | x | x | x | x |
| GCQ | 6-16 |  |  |  |  |  |  |
| QWB |  |  |  |  |  |  |  |

1. The term construct validity is ambiguous, the concept falls under three domains. [↑](#footnote-ref-1)
2. Only scoring if relevant for the Dutch situation, for instance the comparance between a Dutch and Turkish or Moroccan version. [↑](#footnote-ref-2)
3. <https://www.ncbi.nlm.nih.gov/pmc/articles/PMC2859314/> [↑](#footnote-ref-3)
